# The uniformity and stability of cellular mass density in mammalian cell culture

**DOI:** 10.3389/fcell.2022.1017499

**Published:** 2022-10-12

**Authors:** Xili Liu, Seungeun Oh, Marc W. Kirschner

**Affiliations:** Department of Systems Biology, Harvard Medical School, Boston, MA, United States

**Keywords:** cellular mass density, cell volume regulation, mass density homeostasis, cell size control, normalized Raman imaging

## Abstract

Cell dry mass is principally determined by the sum of biosynthesis and degradation. Measurable change in dry mass occurs on a time scale of hours. By contrast, cell volume can change in minutes by altering the osmotic conditions. How changes in dry mass and volume are coupled is a fundamental question in cell size control. If cell volume were proportional to cell dry mass during growth, the cell would always maintain the same cellular mass density, defined as cell dry mass dividing by cell volume. The accuracy and stability against perturbation of this proportionality has never been stringently tested. Normalized Raman Imaging (NoRI), can measure both protein and lipid dry mass density directly*.* Using this new technique*,* we have been able to investigate the stability of mass density in response to pharmaceutical and physiological perturbations in three cultured mammalian cell lines. We find a remarkably narrow mass density distribution within cells, that is, significantly tighter than the variability of mass or volume distribution. The measured mass density is independent of the cell cycle. We find that mass density can be modulated directly by extracellular osmolytes or by disruptions of the cytoskeleton. Yet, mass density is surprisingly resistant to pharmacological perturbations of protein synthesis or protein degradation, suggesting there must be some form of feedback control to maintain the homeostasis of mass density when mass is altered. By contrast, physiological perturbations such as starvation or senescence induce significant shifts in mass density. We have begun to shed light on how and why cell mass density remains fixed against some perturbations and yet is sensitive during transitions in physiological state.

## Introduction

The process of cell size control has recently attracted considerable interest (Ginzberg, Kafri and Kirschner, 2015; Amodeo and Skotheim, 2016; Xie, Swaffer and Skotheim, 2022). Cell size is the outcome of active control of cell growth, coupled to changes in the cell cycle, and reflects changes in metabolism and physiological adaptations to the environment. Studies of cell size in mammalian cells have recently focused on the regulation of cell mass or cell volume (Cadart et al., 2019). Cell volume is generally measured by the Coulter principle or by 3D microscopy, while single cell mass quantification utilizes physical techniques to directly register buoyant or dry mass, rather than wet mass. Cell size and volume have long been known to vary dramatically with cell type. The mass and volume of cells in the human body can vary more than 1000-fold (Ginzberg, Kafri and Kirschner, 2015). By contrast, cellular mass density, which is simply computed by dividing the cell dry mass by the cell volume, has a much narrower distribution than the distributions of either cell mass or cell volume for cells grown *in vitro* and cells in tissues (Bryan et al., 2014; Neurohr and Amon, 2020). In this paper, we use Normalized Raman Imaging (NoRI) to measure single cell’s protein and lipid mass density accurately and directly. We show that cellular mass density for a population of cells is maintained within a remarkably tight distribution when examined for 3 cell lines of different cell types. By perturbing cells mechanically and pharmacologically, we demonstrate the extraordinary stability of cellular mass density, which cannot simply be explained as a passive resultant of mass or volume regulation. Rather it suggests the existence of a homeostatic process of mass density control through active feedback.

The simplest physical model of cell size envisages a bag of impermeable macromolecules bound by a flexible semipermeable membrane. The volume of the bag at steady state would be controlled by osmotic pressure generated by the concentration of impermeable molecules and by the transport of sodium and potassium ions. In this picture, the doubling of the number of impermeant molecules during the cell cycle causes the cell to double both in mass and in volume, hence, maintaining the same mass density. The biophysical picture has been described by a set of flux and constraint equations of the so-called pump leak model (Kay and Blaustein, 2019). The pump leak mechanism predicts that, even without a feedback mechanism, the ratio of cell dry mass to cell volume would be stable, and hence cellular mass density would be constant, provided that the pump rate of ions and the macromolecular and osmolyte composition remain stable during the process of growth. Indeed, cell volume and cell dry mass are thought to be regulated in such a manner in proliferating cells (Cadart et al., 2018; Liu, Yan and Kirschner, 2022). In this process of growth, it is assumed that the cellular molecular composition is unchanging though the total mass changes, thus maintaining the mass density. However, the volume stabilizing behavior of this model is limited to maintaining a steady state mass density independent of size. It may not be able to explain the large variability of cell mass density observed in different cell types, nor does it necessarily explain dynamic cell mass density regulation seen in changing physiological states. Indeed, when cells differentiate or senesce, the composition of the cell changes. The strict parallel of protein content with volume may be abrogated, leading not only to changes in mass but also changes in mass density at steady state. This has been seen in chondrocyte differentiation (Cooper et al., 2013) and in cell senescence (Neurohr et al., 2019; Oh et al., 2022).

It would be ideal if we could accurately measure mass density directly, rather than calculate it by dividing dry mass by volume from independent measurements (Zangle and Teitell, 2014; Cadart et al., 2017; Liu et al., 2020). For very rapid volume changes, it can be assumed that the dry mass does not change appreciably, and mass density could then be calculated from volume change alone (Guo et al., 2017; Roffay et al., 2021; Venkova et al., 2022). But during many physiological changes like differentiation, mass density changes slowly. Under these conditions, it would be unreasonable to assume that there is no change in dry mass (Cooper et al., 2013). Only a few methods can measure mass density directly. The Suspended Microchannel Resonator (SMR) can derive mass density by measuring cell buoyant mass in two different media of different densities, providing sensitivity of 1–6 mg/ml (Grover et al., 2011; Bryan et al., 2014; Miettinen et al., 2022). However, this method is limited to cells grown in suspension and requires medium replacement. Similarly, Quantitative Phase Microscopy (QPM) measures the three variables by measuring the optical path difference of the same cell twice in media of different refractive indices (Cooper et al., 2013). This method also requires medium replacement, and the errors in volume and density measurements are much larger than that of direct dry mass measurement. Refractive index tomography measures local dry mass density in optical cross sections and provides information on the volumetric distribution of subcellular dry mass density (Choi et al., 2007; Kim et al., 2018; Kim and Guck, 2020). However, due to the subtle difference in the refractive index increments of macromolecules, this method has a larger bias in lipid-rich regions and cannot distinguish the contributions of protein and lipid. In part to circumvent these limitations, our laboratory developed Normalized Raman Imaging (NoRI), which has unique advantages for mass density measurements (Oh et al., 2022). It directly and quantitatively measures *mass density* with 15 mg/ml sensitivity in optical z-cross sections (lateral and axial resolutions 0.57 and 1.58 µm) using the stimulated Raman scattering of macromolecules. This principle enables NoRI to separately measure protein and lipid densities of living cells and provides their subcellular localization. The method can also be performed with confluent cultures or in 3-dimensional tissue samples.

In this study, we employed NoRI microscopy on living cultured mammalian cells as a means to measure the protein and lipid mass density directly. We use three mammalian cell lines representing different cell types. We investigate how the mass density responds to perturbations such as extracellular osmotic stress, inhibition of protein synthesis, inhibition of protein degradation, disruption of the cytoskeleton, and other physiological state changes. Though these perturbations have previously been studied extensively for changes in physical and biochemical properties, they had not been compared in terms of changes in cellular mass density. Nor have previous studies distinguished protein from lipid density. We find strong evidence that cellular mass density is under stringent control and is maintained in a remarkably tight range in proliferating cells. It is resistant to some perturbations but can respond to others. These differential responses can help us understand the nature of cell size and mass density regulation during physiological and pathological conditions.

## Materials and Methods

### Cell culture and chemical treatment

HeLa (CCL-2), NIH3T3 (CRL-1658), and RPE-1 (CRL-4000) cells were purchased directly from the ATCC. MDCK II cells were obtained from Jeffrey J. Fredberg laboratory, Harvard T. H. Chan School of Public Health. All cell lines were cultured at 37°C with 5% CO_2_ in Dulbecco’s modified Eagle’s medium (DMEM) (11965; Thermo Fisher Scientific) with 10% fetal bovine serum (FBS) (16000044; Thermo Fisher Scientific), 1% penicillin/streptomycin (15140122; Thermo Fisher Scientific), 25 mM HEPES (15630080; Thermo Fisher Scientific), and 10 mM sodium pyruvate (11360070; Thermo Fisher Scientific), unless indicated otherwise. The starvation medium had the same constitution as the normal medium except 0.1% FBS replaced the standard 10% FBS. The osmolarity of the media was judged to be 350 mOsm using a vapor pressure osmometer (Vapro); all other media were evaluated in the same fashion. The +200 mOsm hyper-osmotic medium was made from normal medium with added 100 mM sodium chloride (S5886, Millipore Sigma), and the +400 mOsm medium was made with added 200 mM sodium chloride; their respective osmolarities were 542 and 742 mOsm. The composition of the hypo-osmotic medium was normal medium diluted with an equal volume of deionized water; it had an osmolarity of 158 mOsm. Rapamycin was purchased from LC Laboratories (R-5000); Cycloheximide, Nocodazole, and Ouabain octahydrate were purchased from Millipore Sigma (C4859, SML1665, and O3125); MG132 and Doxorubicin were purchased from Selleckchem (S2619 and S1208); Cytochalasin D was purchase from Cayman (11330). The NoRI samples were seeded on 55 mm glass bottom dishes with 30 mm micro-well #1.5 cover glass (D60-30-1.5-N, Cellvis), other microscopic samples were seeded on 12 Well or 24 Well glass bottom plates with high performance #1.5 cover glass (P12-1.5H-N and P24-1.5H-N, Cellvis). For trypsinization we used 0.05% trypsin-EDTA (25300054, Thermo-Fisher Scientific) or 0.25% trypsin-EDTA solutions (25200056, Thermo-Fisher Scientific). HeLa and NIH3T3 cells were incubated in 0.05% trypsin-EDTA solution for 10 min and MDCK cells were incubated in 0.25% trypsin-EDTA for 15 min. Trypsinized cells were centrifuged, the supernatant aspirated, and cell pellets were resuspended in a volume of culture medium to achieve the desired seeding density.

### Measurement of the rates of protein synthesis

Protein synthesis rates were assayed by the Click-iT™ Plus OPP Alexa Fluor™ 647 Protein Synthesis Assay Kit (C10458, Thermo Fisher Scientific). Cells were pulse labelled with 10 μM O-propargyl-puromycin (OPP) for 1 h. The Click-iT™ chemistry was carried out according to manufacturer’s instructions. After OPP conjugation with Alexa Fluor™ 647, the cells were stained with 10 μM Hoechst 33342 (62249, Thermo Fisher Scientific) and 500 ng/ml Alexa Fluor™ 568 NHS Ester (SE) (A20003, Thermo Fisher Scientific) for 30 min, followed by two washings with PBS. Cells were then imaged by an Eclipse Ti microscope with the Perfect Focus System (PFS), Plan Fluor 10×/0.3 N.A. PFS dry objective lens (Nikon, Japan), and an ORCA-ER camera (Hamamatsu, Japan). Images were acquired by the NIS-Elements AR ver. 4.13.0.1 software with the WellPlate plugin.

### Assays for cell proliferation

Proliferating cells were detected by the Click-iT™ Plus EdU Cell Proliferation Kit for Imaging, Alexa Fluor™ 647 dye (C10640, Thermo Fisher Scientific). Cells were pulse labeled with 10 μM EdU (5-ethynyl-2′-deoxyuridine) for 1 h. The Click-iT™ chemistry was carried out according to manufacturer’s instructions. After Click-iT™ conjugation, the cells were stained with 10 μM Hoechst 33342 for 30 min and then imaged by fluorescence microscope at 10x magnification.

### Assays for SA-beta-galactosidase activity

SA-beta-galactosidase activity was detected by CellEvent™ Senescence Green Detection Kit (C10850, Thermo Fisher Scientific). Cells were fixed in 2% paraformaldehyde in PBS (diluted from 8% paraformaldehyde, RT 157–8, Electron Microscopy Sciences) for 10 min. The assay was carried out according to manufacturer’s instructions. Cells were stained with 10 μM Hoechst 33342 and 500 ng/ml Alexa Fluor™ 568 NHS Ester for 30 min, followed by two washings with PBS, then imaged by fluorescence microscopy at 10X magnification.

### Measurement of cell size

Cell dry mass was estimated by the SE staining of fixed cells by the following method: cells were fixed with 4% paraformaldehyde for 20 min, permeabilized with 0.5% Triton X-100 in PBS for 20 min, stained with 10 μM Hoechst and 500 ng/ml Alexa Fluor™ 568 NHS Ester (the SE protein dye) for 30 min, and then imaged by fluorescence microscopy. Cell volume was measured by Moxi GO II (Orflo, United States) using the Coulter principle by the following method: live cells were trypsinized by 0.05% or 0.25% trypsin-EDTA, resuspended in DMEM with corresponding drugs or osmotic pressure, and then measured by Moxi GO II using the Cell Count (Size Only) Assay according to manufacturer’s instructions. Debris or dead cells were gated out based on their small diameter.

### Immunofluorescence procedures

Antibodies used in this study are: Anti-S6 Ribosomal Protein (5G10) Rabbit mAb (2217, Cell Signaling), Anti-YAP1 Antibody (63.7) (sc-101199, Santa Cruz), Anti-rabbit IgG (H + L), F (ab')2 Fragment (Alexa Fluor® 488 Conjugate) (4412, Cell Signaling), Anti-mouse IgG (H + L), F (ab')2 Fragment (Alexa Fluor® 488 Conjugate) (4408, Cell Signaling), and Goat anti-Mouse IgG (H + L) Cross-Adsorbed Secondary Antibody, Alexa Fluor™ 647 (A-21235, Thermo Fisher Scientific). During immunofluorescence, cells were fixed with 4% paraformaldehyde for 20 min, then permeabilized and blocked with the blocking buffer (2% BSA, 0.3 M glycine, and 0.1% Tween-20 in PBS) for 1 h. Cells were next incubated with the primary antibody at 1:100 dilution overnight at 4°C, followed by incubation with the secondary antibody at 1:500 dilution for 1 h. Finally, cells were stained with 2 μM DAPI (4, 6- diamidino-2- phenylindole) (D8417; Sigma-Aldrich) and 500 ng/ml Alexa Fluor™ 568 NHS Ester for 30 min, washed three times, and then imaged by fluorescence microscopy at 10x magnification.

To compare cellular mass density and YAP immunofluorescence at the single-cell level, we added a silicon insert confining cells in a small area (81176, ibid) at plating to help locate the imaging area. After NoRI scanning, the cells were immediately fixed and immunostained as described above. The same area imaged by NoRI was found under fluorescence microscopy and imaged at 10X magnification. The fluorescence images of the SE channel were registered to the NoRI images of the protein channel by the Normalized Cross-Correlation (NCC) algorithm (Haralick and Shapiro, 1993).

### Protein and lipid mass density measurements using NoRI microscopy

**FIGURE 1:**
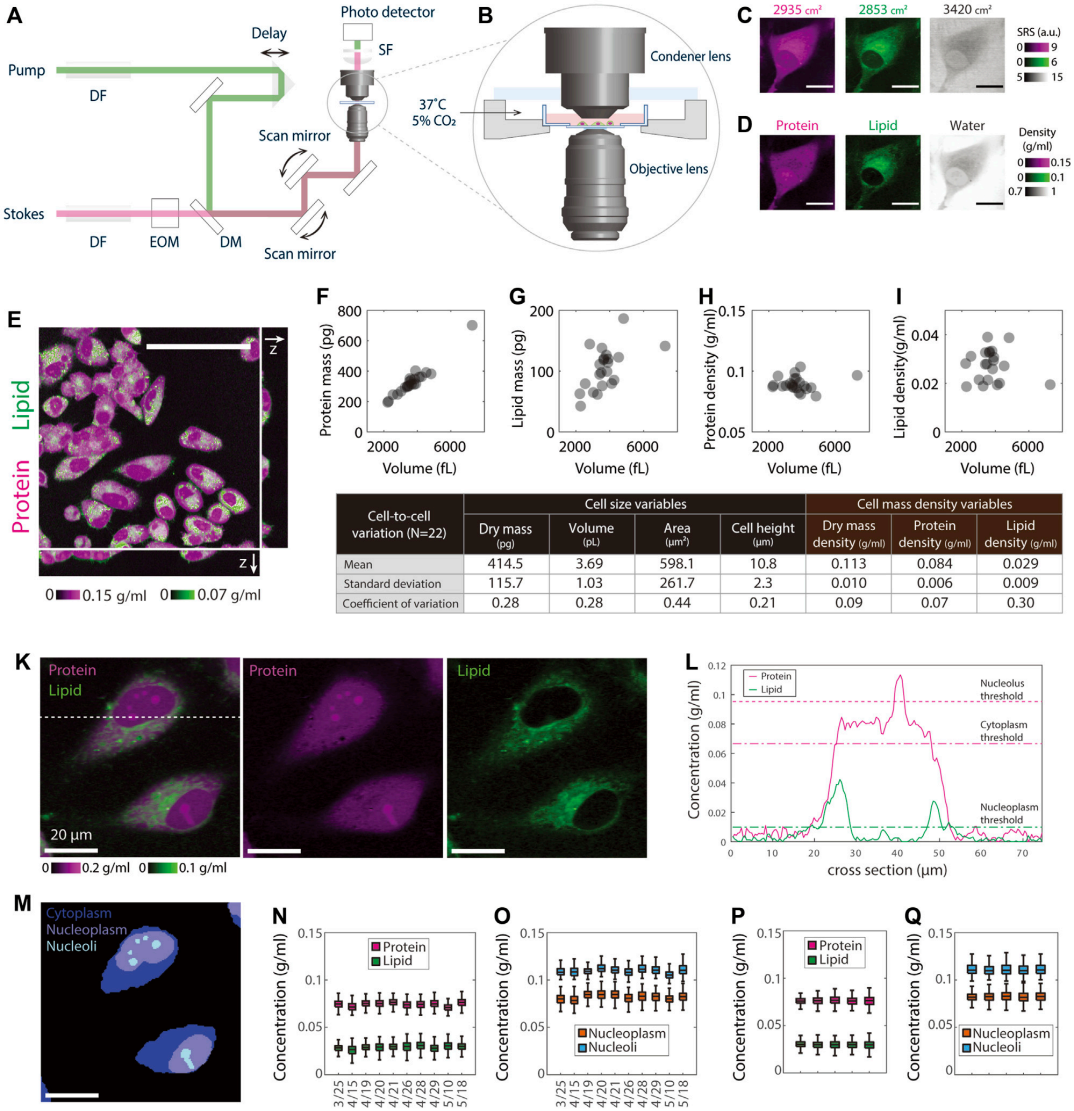
NoRI measurement of single cell mass density demonstrating the stability of protein and lipid mass densities. **(A)** Schematic of the stimulated Raman scattering microscope. DF, dense flint; EOM electro-optical modulator; DM, dichroic mirror; SF, short-pass filter. **(B)** A close-up view of the environmental chamber for live cell NoRI imaging. **(C)** Raw SRS images acquired using the SRS microscope at the Raman bands of 2853, 2935, and 3420 cm^−1^, corresponding to the vibrational modes of CH_2_, CH_3_, and water, respectively. Scale bar, 20 µm. Image intensities are derived directly from the photocurrent at the photodetector. **(D)** The local concentrations of protein, lipid, and water are computed from the raw SRS images by the NoRI algorithm following Oh et al., 2022. **(E)** z-stack NoRI image of fixed A7 cells displaying protein and lipid concentrations. Scale bar, 100 µm. **(F–I)** Correlation plots of protein mass **(F)**, lipid mass **(G)**, protein density **(H)**, and lipid density **(I)** with cell volume measured from single cells (*n* = 22) in **(E)**. The correlation coefficient R is 0.97 (p < 1e-13) for protein mass, 0.56 (*p* = 0.007) for lipid mass, 0.06 (*p* = 0.78) for protein density, and −0.18, (*p* = 0.42) for lipid density, respectively. **(J)** Table summarizing the mean, standard deviation, and CV of the cell size and cell density variables in **(F–I)**. **(K)** Representative NoRI images of live HeLa cells with protein density displayed in magenta and lipid density displayed in green. Scale bar, 20 µm. **(L)** Protein and lipid density profiles along the dashed cross line in **(K)**. The dashed lines indicate the protein (magenta) and lipid (green) thresholds used in the segmentation. **(M)** Segmentation of **(K)** indicating cell bodies, nuclei, and nucleoli. Cytoplasm represents the area inside the cell body outside of the nucleus. Nucleoplasm refers to the area inside the nucleus excluding the nucleoli. **(N–O)** Day-to-day variability of HeLa cell mass density measurements. The average number of cells in each data set is 596 per day. **(P–Q)** Mass densities from five independent cultures of HeLa cells measured on the same day. Mean and standard deviation (cell-to-cell variability) are 76.3 ± 4.8 mg/ml for the cytoplasmic protein, 29.7 ± 5.1 mg/ml for the cytoplasmic lipid, 82.1 ± 5.6 mg/ml for the nucleoplasmic protein and 111.6 ± 10.2 mg/ml for the nucleolar protein. The standard deviation of the mean (sample-to-sample variability) is 0.3, 0.3, 0.4, and 0.8 mg/ml for cytoplasm protein, cytoplasm lipid, nucleoplasm protein, and nucleoli protein, respectively. The average number of cells in each sample is 430.

Protein and lipid concentrations were calculated from stimulated Raman scattering images following the procedure described in our previous report (Oh et al., 2022). Briefly, Stimulated Raman Spectroscopy (SRS) images at 2853 cm^−1^, 2935 cm^−1^, and 3420 cm^−1^ bands (corresponding to the methylene- and methyl-groups, and water characteristic vibrational bands, respectively) were acquired from live or fixed cells using a custom-built spectral-focusing femtosecond SRS microscope. This microscope was constructed using synchronized femtosecond pulse lasers for the Pump and Stokes beams. (Figure 1A). A pair of dense flint (DF) glass rods chirped the pulses. Electro-optical modulator (EOM) modulated the amplitude of the Stokes beam at 20 MHz. A retro-reflector prism mounted on a motorized delay (Delay) was adjusted to control the overlap of the Pump and Stokes beams. The pump and Stokes beams, combined by a dichroic mirror (DM), were focused on the sample by the objective lens. Images were acquired by point-scanning by a pair of galvanized mirrors (scan mirror). After passage through the stimulated Raman scattering at the sample plane, the Pump beam was collected by a high numerical aperture condenser lens, selected by a short pass filter (SF), and its intensity was measured by a high-speed photodetector. Cells were maintained at 37°C in 5% CO_2_ during imaging using TomoChamber stage-top incubator (Tomocube, South Korea) with a custom-fitting adapter for the immersion condenser. (Figure 1B). The three SRS images were spectrally unmixed into protein, lipid, and water components using reference spectra measured from bovine serum albumin solution in water, dioleoyl-phosphocholine solution in per-deuterated methanol, water, and per-deuterated methanol. Unmixed images of protein, lipid, and water components were converted to the absolute concentration by using the sum of the three components as the normalization reference at each pixel. Dry mass density was calculated from the sum of protein mass density and lipid mass density.

### Cell cycle determination with NoRI

To determine cell cycle position of single cells along with their NoRI mass density measurement, we stained live cells with 2 μM Hoechst 33342 and acquired fluorescence images using the confocal microscope embedded in the NoRI microscope (Olympus FV3000, excitation at 405 nm) at 10x magnification with a fully opened confocal pinhole. The field illumination was corrected by a fluorescence reference slide (2273, Ted Pella). The large field Hoechst fluorescence image at 10x magnification was registered to the NoRI image mediated by a Hoescht image taken at the same magnification (60x) as the NoRI image.

### Data analysis

All images were processed by customized codes in Matlab (Mathworks) or ImageJ (National Institute of Health). Single cells, nuclei, and nucleoli in NoRI images were automatically segmented by thresholding using protein densities greater 0.0666 g/ml to define the cell body and lipid densities of less than 0.0099 g/ml for the nucleus. Nucleoli were segmented by applying Otsu’s method (Otsu, 1979) on protein density within the nucleus. Morphological operation were used to select the relevant features by size thresholds. The watershed algorithm was used to draw the boundary of adjacent cells in confluent culture. See accompanying online materials for the Matlab codes. Statistical analyses were performed by customized codes in Matlab. Samples were compared by the one-way ANOVA test, and N.S., *p* > 0.05; *, *p* < 0.05; **, *p* < 0.01; ***, *p* < 0.001; ****, *p* < 0.0001 were used to denote the statistical significance. All measurements reported in this manuscript were repeated more than once to confirm reproducibility. However, in the figures we chose to display the data from single experiments rather than pooling multiple datasets to avoid noise introduced by day-to-day variability. This improves the visibility of data trends.

## Results

### Protein and lipid densities measured by NoRI are strikingly consistent within cells of each cell type

To measure the density of protein and lipid in subcellular compartments of live cells in absolute terms, we turned to Normalized Raman Imaging (NoRI), which we recently described (Oh et al., 2022). NoRI enables a label-free direct measurement of protein, lipid, and water density in optical sections of live or fixed cells; total mass can be calculated from a z-stack image by integrating the mass density over cell volume. In brief, this method works as follows: Near-infrared pulse lasers at the pump and Stokes wavelengths were combined and scanned through the sample using a point scanning microscope (Figure 1A); The intensity of the Stokes beam was modulated using an electro-optical modulator (EOM), and the stimulated Raman loss (SRL) of the transmitted pump beam was measured by a photo detector; Live cell samples were maintained at 37°C under 5% CO_2_ atmosphere (Figure 1B); The SRL signal was demodulated by a lock-in amplifier at the EOM modulation frequency to obtain the SRS images at whichever Raman band is selected by the energy difference between the pump photons and Stokes photons (Figure 1C); A “NoRI algorithm” computes the absolute concentrations of protein, lipid, and water from the SRS intensities at 2935, 2853, and 3420 cm^−1^ Raman bands, which correspond to the vibrational modes of CH_3_ groups, CH_2_ groups, and water molecules, respectively (Figure 1D). In this scheme, the mass of nucleic acids is not separately measured but is added to protein mass after significant fractional reduction, since the nucleotide absorption only slightly overlaps the protein peak (Oh et al., 2022; details in Discussion). This is only a small correction since the mass concentration of nuclei acids is much smaller than that of protein in mammalian cells (Oh et al., 2022); therefore the protein mass measured in this manuscript is predominantly from proteins, not nucleic acids. In other circumstances where this is important, for example, in mitotic chromosomes, we can separately measure nucleic acids, protein, lipid, and water by the 4-band NoRI (Oh et al., 2022).

In the following assay, we ask what the most stringently regulated parameters of cell size are by comparing the coefficients of variations of cell volume, cell dry mass, and cellular mass density. Furthermore, we ask this question by separately considering the mass and density of protein and lipid. We initially assessed the relationship between cell dry mass and cell volume in A7 cells, a human cell line that was originally derived from a malignant melanoma (Figure 1E), by integrating the z-stack of NoRI images. To avoid any issues of phototoxicity in living cells, we used fixed cells in this experiment. To identify the contours of the cell, we thresholded the z-stack images of protein density of roughly 1 µm optical sections, from which we obtained the cell volume segmentation. The integration of protein or lipid density over the cell volume constituted the total protein or lipid mass; the ratio of mass to cell volume represented the averaged protein or lipid density of the cell. Not surprisingly, we found that both total protein and lipid mass linearly scaled with cell volume (R = 0.97 and 0.56, respectively) (Figures 1F,G). Notably, protein and lipid densities were nearly completely independent of cell volume (R = 0.06 and R = 0.01, respectively) (Figures 1H,I). The variability of cells in the population, as quantified in terms of the coefficient of variation (CV), was 28% for both cell volume and cell dry mass (the sum of protein and lipid mass). If cell volume and cell dry mass were independent variables, the CV of mass density should be equal to or greater than the combined CVs of the two. However, the observed CV of cellular mass density (the sum of protein and lipid density) was only 9%, and the CV of protein mass density was only 7%, much smaller than either the CV of cell dry mass or the CV of cell volume. We conclude that there must be tight coordination of cell dry mass and cell volume in individual cells (Figure 1J). The CV (30%) of lipid mass density was much higher than that of protein mass density. The much weaker correlation between lipid *content* and cell volume may reflect very different regulatory circuits for lipid and protein mass regulation (Alberts et al., 2002).

To carry out measurements in live cells, we took the NoRI image of the cells only at the midsection. We did this for two purposes: to avoid phototoxicity from extensive 3D scanning and as a strategy to greatly increase the number of cells we practically could measure. We verified that the protein and lipid density measured from a single z-plane showed excellent agreement with those averaged over the whole cell volume (Supplementary Figures S1A,B). We estimated the measurement error of protein density in a single cell cross-section as the following: The measurement sensitivity of the protein channel is 15 mg/ml (Oh et al., 2022), and the cell area at the mid-section is about several hundred pixels at 60x magnification; As the error decreases with the squared root of the number of pixels measured, the error of protein density averaged in one section is less than 1.5 mg/ml; As the cell protein density is about 80 mg/ml in cultured cells, the measurement error for protein density is less than 2%. Similarly, we estimated the lipid measurement error, which is less than 5%. Both the measurement errors of protein and lipid densities were much less than the cell-to-cell variation we observed in total, protein, and lipid mass densities (Figure 1J). For this reason, we felt confident quantifying cell protein and lipid densities from single NoRI cross sections in live cells, an approach we used for the rest of this manuscript.

To process the large number of images required to generate statistically significant results, we developed automated segmentation algorithms for single cells and organelles (Figures 1K–M, Supplementary Figures S1I–K). Benefiting from the remarkable homogeneity in cytoplasmic protein density and its sharp drop at the cell edge (Figure 1L, Supplementary Figure S1J), we were able to apply a universal protein density threshold to detect the boundary of the cell bodies. Since nuclei have much lower lipid density than cytoplasm, they can be easily segmented as the area inside the cell body that has the lipid density smaller than a lipid threshold. Nucleoli were segmented as denser areas within nucleus by the Otsu’s thresholding method (details see Material and Methods: Data Analysis). Such segmentation algorithms allowed us to process efficiently the NoRI images of different cell lines under all the different perturbations using a common objective approach without the use of additional segmentation markers or the need for subjective adjustment of the detection thresholds. Throughout the rest of this manuscript, we characterized protein and lipid densities in three subcellular compartments: the cytoplasm, nucleoplasm, and nucleolus (Figure 1M). Since the lipid density is very low in the nucleus (an average of 5 mg/ml), we analyzed the lipid density only in the cytoplasm.

To assess the reproducibility of the density measurements by NoRI, we measured the protein and lipid density in live HeLa cell cultures over a period of 7 weeks. This day-to-day variability should encompass all the instrumental variation, the variability introduced in the instrument calibration process, any biological variation introduced by using different aliquots of culture media and serum, and variation caused by cell passage number, plating density, and fluctuations in the environment. We found all the cytoplasmic, nucleoplasmic, and nucleolar protein and cytoplasmic lipid densities were maintained within tight ranges (Figures 1N,O). Consistent with the fixed cell measurements (Figure 1J), the cytoplasmic protein density was 74.5 ± 5.2 mg/ml (CV = 0.07), the cytoplasmic lipid density was 29.1 ± 5.3 mg/ml (CV = 0.18). In addition, the nucleoplasmic protein density was 81.9 ± 5.4 mg/ml (CV = 0.07), and the nucleolar protein density was 110.0 ± 5.9 mg/ml (CV = 0.05). Day-to-day variability of daily mean values showed standard deviations of 1.7 mg/ml (CV = 0.02) for the cytoplasmic protein, 1.5 mg/ml (CV = 0.05) for the cytoplasmic lipid, 2.2 g/ml (CV = 0.03) for the nucleoplasmic protein, and 2.1 mg/ml (CV = 0.02) for the nucleolar protein, which was less than half of the corresponding cell-to-cell variability. Biological replicates measured on a single day (Figures 1P,Q) showed even less variability (CV = 0.004–0.009), demonstrating both the excellent repeatability of our NoRI measurements and the remarkable stability of protein and lipid densities in HeLa cells.

### Protein densities are maintained in tight ranges in each of the cell lines investigated

**FIGURE 2:**
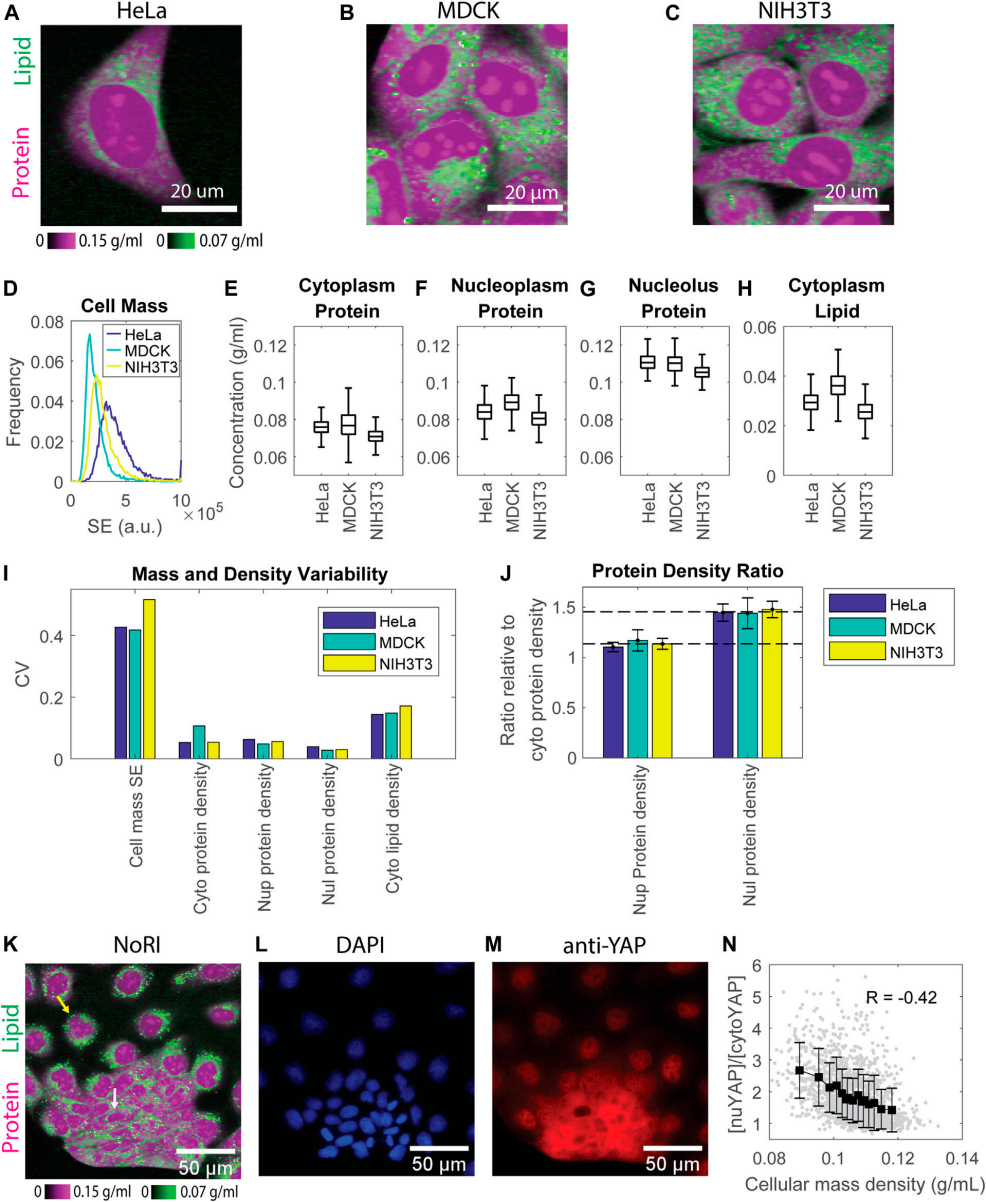
Protein densities are maintained within tight ranges in all 3 cell lines investigated. **(A–C)** NoRI images of HeLa **(A)**, MDCK **(B)**, and NIH3T3 **(C)** cells. **(D)** Dry mass distribution of the 3 cell lines estimated by the SE protein stain. *n* = 9019 for HeLa, 14478 for MDCK, and 12892 for NIH3T3, respectively. **(E–H)** Protein concentration in the cytoplasm **(B)**, nucleoplasm **(C)**, nucleolus **(D)**, and lipid concentration in the cytoplasm **(E)** measured by NoRI. *n* = 1570 (HeLa), *n* = 3056 (MDCK), *n* = 2330 (NIH3T3). **(I)** CVs of dry mass and mass densities in **(D–H)**; Nup, nucleoplasm; Nul, nucleolus; Cyto, cytoplasm. **(J)** The ratio of nucleoplasm (Nup) or nucleolus (Nul) protein density to cytoplasm (Cyto) protein density. The long dashed line indicates the averaged nucleoplasm to cytoplasm protein density ratio of the 3 cell lines at 1.1. The short dashed line indicates the averaged nucleolus to cytoplasm protein density ratio of the 3 cell lines at 1.45. **(K)** NoRI image of MDCK cells showing the heterogeneous morphology: the yellow arrow indicates a representative spread-out cell, and the white arrow indicates a representative compact cell **(L,M)** Fluorescence images of DAPI stained nuclei **(L)** and immunostained YAP **(M)** in the same FOV as **(K)**. **(N)** YAP localization, quantified as nuclear YAP mean intensity divided by cytoplasmic YAP mean intensity, versus cellular mass density. Each grey dot represents a cell; black squares are the mean of each bin; error bars indicate the standard deviation of the bin; R is Pearson’s correlation.

To test the pump leak model’s prediction of that mass density is independent of cell dry mass, we quantified protein and lipid density in three different mammalian cultured cell lines: HeLa, as a representative cancer cell line from human, MDCK II as a representative epithelial cell line from dog, and NIH3T3 as a representative fibroblast cell line from mouse (Figures 2A–C). We chose these different cell types with their divergent genetic backgrounds so that any consistent behavior of mass density observed among the three would suggest that it could be a universal, conserved property of cultured, proliferating mammalian cells.

Since protein constitutes more than 70% of cell dry mass (Alberts et al., 2002), we estimated the total dry mass in these cell lines by the SE protein dye (Kafri et al., 2013) and quantified the protein and lipid densities by NoRI. The SE staining is specific for lysine groups which are nearly all on the surface of proteins and should not respond to conformational changes or denaturation. Thus, the SE modification of proteins should be stoichiometric or nearly stoichiometric (Kafri et al., 2013). However, we are aware that there could be some potential limitation of SE staining when comparing different cell lines or the same cell line under different conditions, which might express proteins with different lysine content. Nevertheless, in our measurements of different cell lines, we found that the difference in mass density was much smaller than the difference in cell dry mass. Among the 3 cell lines, HeLa is the most massive, and MDCK is the least. Their mean dry mass difference is 1.9 fold (Figure 2D). By contrast, the densest cell line, MDCK, is only 1.2 fold more dense than the most diluted cell line, NIH3T3. The difference in protein density in all three compartments is no larger than 1.1 fold (Figures 2E–G). Cytoplasmic lipid density is more variable, with the difference between the densest and most dilute cell lines being 1.4 fold (Figure 2H). These results suggest that cultured mammalian cells maintain mass density in a much narrower range than they maintain their dry mass. Furthermore, in each cell line, the protein densities in all three compartments were maintained in tight ranges. The cell-to-cell variability (CV) was 5%–11% for cytoplasm, 5%–6% for nucleoplasm, and 3%–4% for nucleolus protein density, respectively. The variability of cytoplasm lipid density was higher, with 14%–17% CV (Figure 2I).

Consistently among all 3 cell lines, protein density in the nucleolus was higher than that in the nucleoplasm, which was higher than that in the cytoplasm (Figures 2E–G). The ratio of protein density in the three compartments is very close among the 3 cell lines, with 1.5:1.1:1 in HeLa, 1.4:1.2:1 in MDCK, and 1.5:1.1:1 in NIH3T3 cells (Figure 2J). The much denser nucleolus than other cell compartments is consistent with published measurements of cellular mass density by refractive index (Kim and Guck, 2020). The cellular mass density (the sum of protein and lipid density) ratio of these three compartments was around 1:0.85:1.05 (Supplementary Figure S2), close to the 1:0.8:1.2 ratio found in chick nerve cells originally measured by interference microscopy (Merriam and Koch, 1960). Merriam et al. found that the ratio was constant during chick embryo development. We now find that it is also constant in three different cell lines of different cell types and genetic backgrounds, raising the question of whether the ratio might be a universal biophysical property of mammalian cells and under which conditions it might be perturbed. Note that in the 3-component NoRI method, nucleic acid mass was attributed to protein mass due to its partial overlap with the Raman band of proteins at 2935 cm^−1^. In fixed HeLa cells, it caused 8 ± 2 mg/ml (12 ± 3%) and 11 ± 3 mg/ml (19 ± 5%) increment in protein density in the interphase nucleus and condensed chromatins in mitotic cells, respectively (Oh et al., 2022). Taking this into consideration, the protein densities in the nucleoplasm and cytoplasm are very close, consistent with the indistinguishable nuclear and cytoplasmic densities in fission yeast (Odermatt et al., 2021).

Although protein densities are maintained in a tight range for each of the 3 cell lines, we noted that MDCK cells had a nearly 2-fold higher variation in cytoplasmic protein density than HeLa and NIH3T3 cells (CV 10.6% in MDCK vs. CV 5.3% in HeLa and NIH3T3). A closer look at MDCK NoRI images revealed obvious heterogeneity in the cell morphology within the culture. There were two distinct groups of cells: one group was more spread-out and dilute, whereas the other group appeared more compact and dense (Figure 2K). Their cellular mass density was only weakly correlated with their cell dry mass (Pearson’s correlatio4 0.08, *p* = 0.002) (Supplementary Figure S3A), suggesting that the major difference between the groups was in their volumes. Since YAP (the key transcriptional cofactor in the Hippo pathway) has been linked to cell volume regulation (Gonzalez et al., 2018; Perez-Gonzalez et al., 2019), we investigated the distribution of YAP protein by immunofluorescence in the MDCK cells. YAP is stable and can act as a transcription factor when it is in the nucleus, but it is subjected to degradation when it is translocated to the cytoplasm, where it is phosphorylated (Pocaterra, Romani and Dupont, 2020; Kwon, Kim and Jho, 2021). We found that YAP was primarily localized to the nucleus in the cells with a spread morphology, whereas in cells with a compact morphology YAP was primarily present in the cytoplasm (Figures 2K–M). To express this observation more quantitatively, we denoted the YAP localization by the ratio between the mean intensities of nuclear and cytoplasmic YAP. We found a strong correlation between that ratio and the cellular mass density (Pearson’s correlation = −0.42, *p* < 1e^−66^) (Figure 2N). However, the total YAP intensity was only weakly correlated to the cellular mass density (Pearson’s correlation = −0.05, *p* = 0.03) (Supplementary Figure S3B). Therefore, the mass density depends more on YAP localization than the level of YAP expression. These results suggest a functional connection between cellular mass density and YAP localization. However, at this point, we cannot conclude that YAP activity is actually higher in dilute cells. Since dense cells have the same mass as dilute cells (Supplementary Figure S3A), they also must have a smaller volume, and the observed density increase could mean that the concentrations of all proteins are higher. We cannot assume this as different proteins could be differently affected. However, this may be the reason that the YAP concentration is higher in both the nucleus and cytoplasm of denser cells. (Supplementary Figures S3C,D). The N/C ratio was also higher in dense cells, resulting in more YAP molecules in the nucleus (Supplementary Figures S3E,F). Thus, YAP’s partition between nucleus and cytoplasm and its absolute concentration in these two compartments point to different directions of YAP regulation: the partition suggests it is downregulated in denser cells, but the concentrations suggest the opposite. A firm mechanistic understanding of the relationship of YAP transcriptional activity and YAP localization to mass density will require further experiments.

### Mass density does not vary appreciably in the cell cycle

**FIGURE 3:**
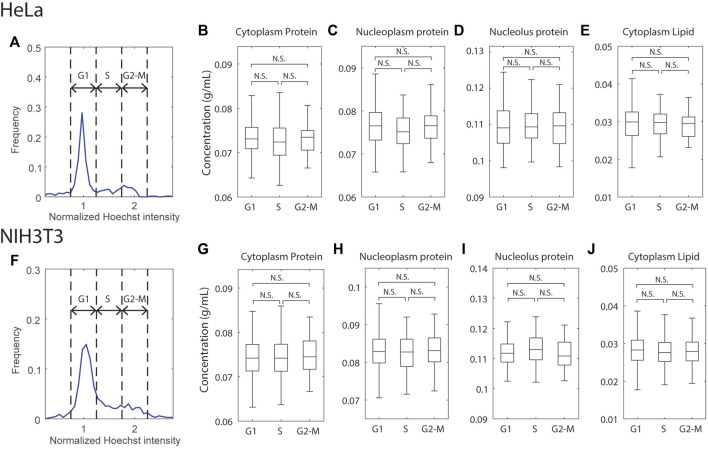
Mass densities are independent of the cell cycle. **(A,F)** Histogram of total Hoechst intensity in the nucleus in cycling HeLa **(A)** or NIH3T3 **(F)** cells; the Hoechst intensity was normalized to the G1 peak of the distribution. Dashed lines indicate the gates for G1, S, and G2-M cells. **(B–E,G–J)** Mass densities in HeLa **(B–E)** and NIH3T3 **(G–J)** cells, grouped by the cell cycle stages. *n* = 250 (HeLa G1), 53 (HeLa S), 53 (HeLa G2-M), 392 (NIH3T3 G1), 123 (NIH3T3 S), 95 (NIH3T3 G2-M).

To ask whether mass densities change during the cell cycle, we used Hoechst staining to monitor DNA content and from that, infer cell cycle stage in live cells. The cells were imaged on the confocal optics embedded in the NoRI microscope to quantify Hoechst intensity; we acquired images of protein and lipid mass densities with NoRI (see Material and Methods for more details). In this manner, we were able to identify the cell cycle stage and mass density in the same cell. The confocal pinhole was fully open to capture the Hoechst signal from the entire height of the nucleus. Due to the fact that MDCK cells are very tall, as measured from the dish to their apex, we were only able to quantify DNA content in HeLa and NIH3T3 cells (Figures 3A,F).

There was a small complication in NoRI imaging with Hoechst-stained cells. Hoechst staining is accompanied by a decrease in protein signal and an increase in lipid signal (Supplementary Figure S4). The effect is especially pronounced in the nucleoplasm protein density, where it caused a 3.7 mg/ml (4.5%) decrease in HeLa and a 4.2 mg/ml (4.8%) decrease in MDCK cells, respectively. Hoechst dye might affect mass density quantification in two ways. First, two-photon absorption (TPA) of Hoechst would add non-specific background to the SRS signals, which, after spectral decomposition and normalization, could artificially increase the lipid density and decrease the protein density. Second, most Hoechst molecules carry a positive charge at the intracellular pH (Swain et al., 2020). When Hoechst localizes to the nucleoplasm, their counter ions can increase the osmotic pressure inside the nuclear envelope and thus decrease nuclear mass density. At saturation, Hoechst can cause a 1–2 mg/ml artificial decrease in nucleoplasmic protein density quantified by NoRI and add somewhat less than 5 mOsm osmotic pressure to the nucleus. The two effects combined most likely generated the observed small density changes seen with Hoechst stain. Nevertheless, because the nuclear volume increases with the cell volume during cell cycle, the DNA (or Hoechst) concentration (not content!) is independent of the cell cycle except for mitotic cells. Therefore, we assume that Hoechst stain does not appreciably change the relative differences in protein and lipid mass densities at different cell cycle stages.

With these considerations in mind, we binned cells at the G1, S, and G2-M stages by their total Hoechst intensity in the nucleus and found no significant difference in protein or lipid density in any of the compartments between any 2 cell cycle stages in either HeLa or NIH3T3 cells (Figures 3B–E,G–J). This result is consistent with the previous density measurement by refractive index (Kim and Guck, 2020) but contrasts with measurements of the cell cycle-dependent molecular crowding (Lecinski et al., 2021; Yamamoto et al., 2021) and diffusion rate (Pradeep and Zangle, 2022). Although mass density, molecular crowding, and diffusion rate are related, they are evaluated by molecules of very different size, which may result in distinct behaviors. For example, the cytoskeleton will perturb movement/diffusion of large macromolecular diffusion probes while being invisible to smaller ones. It is known that mitotic cells swell and dilute their mass density in prophase and prometaphase (Son et al., 2015; Zlotek-Zlotkiewicz et al., 2015), which we have confirmed with NoRI (Oh et al., 2022). However, we did not observe noticeable decreases in the G2-M densities. This may be because swollen mitotic cells only constitute a small fraction of the G2-M cells, and our automatic segmentation code has difficulty segmenting the mitotic cells as they do not have a recognizable nucleus. To conclude, the NoRI measurements are consistent with the expectations of the pump leak model, where mass density is conserved when applied to proliferating cells. In the pump leak model, mass density is maintained at a constant value because dry mass itself is the principal regulator of cell volume. Therefore, this relationship between dry mass and volume maintains mass density through the cell cycle, even though DNA content, cell dry mass, and cell volume double.

### How external osmotic stress affects cytoplasmic density

**FIGURE 4:**
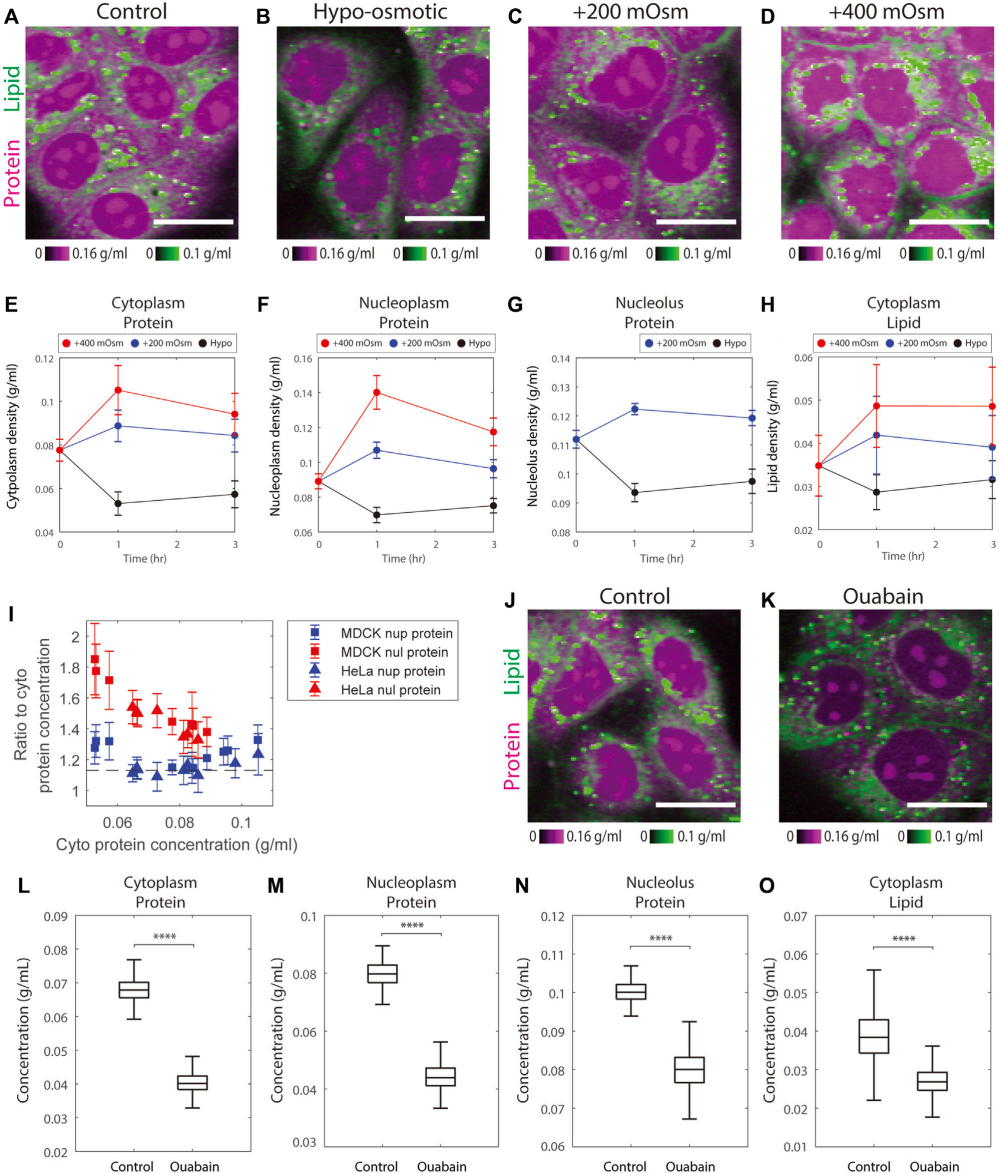
Osmotic stress alters protein and lipid densities. **(A–D)** Representative NoRI images of control MDCK cells in complete medium and MDCK cells after 3 h in hypo-osmotic or hyper-osmotic media. Scale bar, 20 µm. **(E–H)** Time course of protein and lipid density change by hyper-osmotic and hypo-osmotic treatment. Time 0 shows the control sample. Data points and error bars are the mean and standard deviation. The number of cells in each data point is between 452 and 1204 cells, with a mean of 790 cells. **(I)** The ratio of nucleoplasm (nup) or nucleolus (nul) protein density to cytoplasm protein density versus cytoplasm protein density measured in the control, hypo-, and hyper-osmotic media. The dashed line indicates the mean ratio between nucleoplasm and cytoplasm protein density from Figure 2J. **(J–K)** Representative NoRI images of control MDCK cells and MDCK cells treated with 3 µM ouabain for 5 h. Scale bar, 20 µm. **(L–O)** Change of protein and lipid density in cytoplasm, nucleoplasm, and nucleolus in MDCK cells treated with 3 µM ouabain for 5 h. *n* = 548 (Control).*n* = 665 (Ouabain).

According to both the simple van’t Hoff equation (Atkins and De Paula, 2006) and the more sophisticated pump leak model of Essig (Essig, 1968), external osmotic stress has a direct effect on cell volume. Since a cell’s response to such an external osmotic force is nearly instantaneous, it has usually been assumed that cell dry mass does not change and that any change in mass density can be directly attributed to the change in cell volume. To evaluate whether this assumption holds, we measured both the effect of external osmotic stress on intracellular density, cell mass, and cell volume. Specifically, we measured the mass densities in MDCK and HeLa cells at 1 and 3 h after switching to hypo-osmotic or hyper-osmotic media. The hypo-osmotic medium used in this study was made by diluting the complete medium with de-ionized water, and the hyper-osmotic media was by adding additional sodium chloride. As expected, cytoplasmic protein and lipid densities decreased in hypo-osmotic medium and increased in hyper-osmotic media (Figures 4A–E,H, Supplementary Figures S5A–E,H). The magnitude of the density shift decreased from 1 h to 3 h, perhaps because the cells may have changed their composition in response to the osmotic shock and/or because the initial osmotic volume change was counteracted by regulatory responses in the direction of restoring the initial volume. However, the density changes could not be solely attributed to changes in cell volume. Even a 1 hour exposure to hypo-osmotic medium caused a sizable dry mass increase (21%) in MDCK cells. The surprisingly large dry mass increase may be partially due to the compositional change of cell proteome, resulting in a disproportional change in the SE stain. Despite the dry mass increase, the volume increase was more dramatic (35%) (Supplementary Figure S6). Together, they accounted for the observed but nevertheless muted mass density decrease.

Nucleoplasmic protein density changes matched the changes in cytoplasmic protein density; the ratio of the two remained constant (Figure 4I, Supplementary Figure S7A). This behavior suggests that cells rapidly adjust their nuclear volume, perhaps in response to pressure exerted on the nuclear envelope during the osmotic response. This explanation is consistent with the previous findings that nuclear size is controlled by osmotic force within the cell (Finan and Guilak, 2009; Deviri and Safran, 2022; Lemière et al., 2022). Though nucleolar protein density changed in the same direction as nucleoplasmic protein density, the amplitude was smaller (Figure 4I, Supplementary Figure S7A). Therefore, in the hypo-osmotic medium the protein density in nucleoli became even more distinct from that of nucleoplasm, whereas in hyper-osmotic media, the protein densities in the two compartments were similar. Particularly in the +400 mOsm medium, the protein densities in the two compartments were so close that the nucleoli became indistinguishable from nucleoplasm. As a consequence, we were no longer able to segment nucleoli using protein mass density differences (Figure 4D, Supplementary Figure S5D). The protein densities of nucleolus and nucleoplasm were 0.12–0.14 g/ml in the +400 mOsm hyper-osmotic medium, a density, that is, still far below the upper limit of protein compaction (the cytoplasmic protein density of bacteria or red blood cells are over 0.3 g/ml). This suggests that the slow increase of nucleus protein density in hyper-osmotic media was not limited by protein compaction. The behaviors of nucleolus protein density in hypo- and hyper-osmotic media are consistent with the theory that nucleoli are formed by liquid-liquid phase separation generating a biomolecular condensate (Feric et al., 2016; Lafontaine et al., 2021); such a condensate would not be expected to respond to osmotic forces in the same way as the nucleoplasm, which behaves more as a classical solution.

In iso-osmotic conditions, cell volume is thought to be maintained by ion channels and transporters at the plasma membrane through the pump-leak mechanism (Essig, 1968). As the model predicts, when we inhibited the sodium-potassium pumps by ouabain for 5 h, there was a dramatic decrease in the protein densities of the cytoplasm, nucleoplasm, and nucleoli to 0.6, 0.6, and 0.8 times their original densities, respectively, as compared to control cells (Figures 4J–N). Cytoplasmic lipid density also decreased by 0.7 fold times its original density (Figure 4O). These changes were consistent with the previously observed 35% volume increase in MDCK cells treated with ouabain for 5 h (Platonova et al., 2011). In short, the results of cells treated with hypo- or hyper-osmotic media or ion pump inhibitors demonstrate the expected direct effect of osmoregulation on mass density.

### Cytoskeleton disruption leads to slight increases in protein density

The pump leak model assumes that the plasma membrane maintains little tension and that it is very compliant in expansion and contraction (Kay, 2017). If the plasma membrane cannot exert much tension, perhaps the cytoskeleton can absorb some of the osmotic forces (Sitarska and Diz-Muñoz, 2020). How much the contractile and/or tensile forces of the cytoskeleton contribute to cell volume regulation has not been fully resolved (Tao and Sun, 2015; Guo et al., 2017; Kim and Guck, 2020; Venkova et al., 2022). The uncertainty that exists in the literature may be because membrane tension is specific to cell type, cell shape, and interactions with the extracellular matrix (Le Roux et al., 2019). To account for contributions of the cytoskeleton (Venkova et al., 2022). further extended the classic pump leak model by adding the mechano-sensing factor, which would act to change of membrane tension by modulating the activity of ion pumps thus changing mass density (Venkova et al., 2022). We investigated the effect of cytoskeleton on mass density by treating HeLa and MDCK cells with cytoskeleton depolymerizing drugs and with trypsin, which would act to release cells from the extracellular matrix.

**FIGURE 5:**
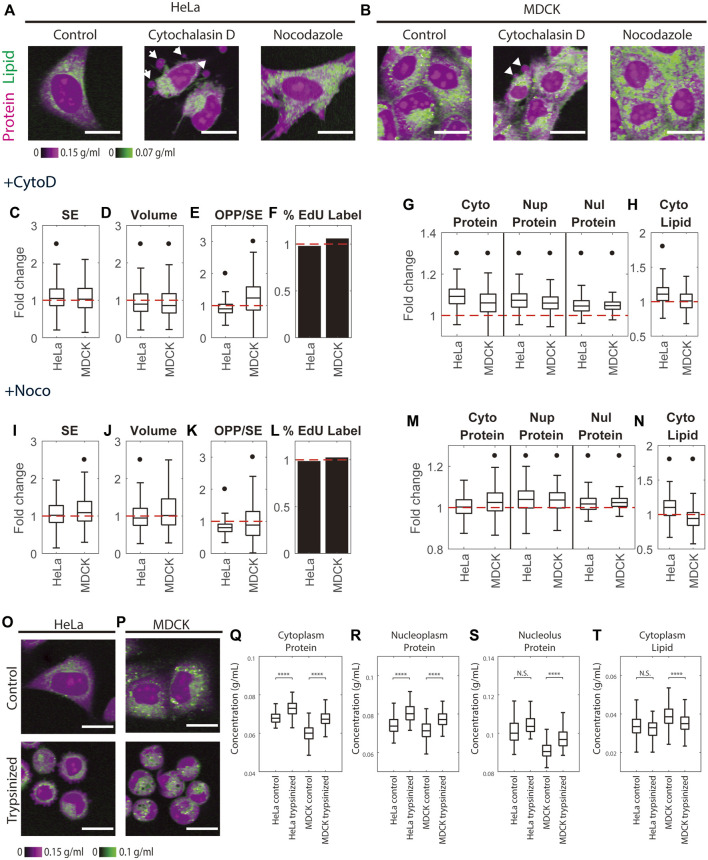
Cytoskeleton perturbation increases protein density. **(A–B)** Representative NoRI images of untreated HeLa and MDCK cells and cells treated with 5 µM Cytochalasin D or 5 µM Nocodazole for 1 h. White arrows indicate blebs. Scale bar, 20 µm. **(C–F, I–L)** Fold change of cell dry mass (SE) **(C,I)**, cell volume **(D,J)**, protein synthesis rate quantified by the pulse labeled OPP to SE protein stain ratio (OPP/SE) **(E,K)**, and DNA replication (percentage of EdU labeled cells) **(F,L)** in Cytochalasin D treated (+CytoD) or Nocodazole treated (+Noco) cells normalized to the median of untreated cells. **(G–H, M–N)** Fold change of protein densities and cytoplasmic lipid density in Cytochalasin D treated **(G–H)** or Nocodazole treated **(M–N)** cells normalized to the median of untreated cells. Red dashed lines in **(C–N)** indicate fold change = 1 (no change). Black dots in **(C–N)** denote significant changes compared to control. The absolute changes in **(C–N)** are summarized in Supplementary Figures S8. **(O,P)** Representative NoRI images of HeLa **(O)** and MDCK **(P)** cells before and 1 h after trypsinization. **(Q–T)** Cytoplasmic protein density **(Q)**, nucleoplasm protein density **(R)**, nucleolus protein density **(S)**, and cytoplasm lipid density **(T)** in pre-trypsinized (*n* = 213) and post-trypsinized (*n* = 108) HeLa cells and pre- trypsinized (*n* = 578) and post-trypsinized (*n* = 313) MDCK cells.

Cytochalasin D is an actin depolymerizer that acts at the level of the actin subunit (Schliwa, 1982). Nocodazole is widely studied as directly acting on the tubulin dimer and blocks polymerization. Its limited toxicity and rapid reversibility have led to its wide use as an easily reversible cell cycle blocker (Downing, 2000). The effects of both drugs on cells are very rapid. Therefore, we needed to treat cells with these drugs for 1 h to massively depolymerize the cellular actin and tubulin. Neither drug caused a significant change in DNA replication (Figures 5F,L). We expected if there were any changes in mass density caused by these drugs, it should be due to a change in cell volume, not in cell dry mass. However, we found small yet significant mass *increases* in Hela treated with Cytochalasin D and MDCK treated with Nocodazole (5 and 9%, respectively) (Figures 5C,I). Cytochalasin D also caused a 10%–14% volume decrease in HeLa and MDCK cells (Figure 5D). Nocodazole caused a 7% volume decrease in HeLa but no significant change in MDCK cells (Figure 5J). The increase of cell dry mass and decrease of cell volume together caused mass density elevation in cells treated with either of the drugs. Cytochalasin D caused a 6%–9% increase in cytoplasmic protein density, a 6%–7% increase in nucleoplasmic protein density, and a 5% increase in nucleolar protein density (Figure 5G). The effect of Nocodazole was less pronounced (Figure 5M). It caused a 0%–3% increase in cytoplasmic protein density, a 4% increase in nucleoplasmic protein density, and a 2% increase in nucleolar protein density. The effects of the drugs on cytoplasmic lipid density were inconsistent among cell lines (Figures 5H,N), with a 10% increase in HeLa treated with Cytochalasin D or Nocodazole and a 6% decrease in MDCK treated with Nocodazole. The change in MDCK cells treated with Cytochalasin D was insignificant. Overall, we observed a slight protein density increase (no greater than 9% in Cytochalasin D and no greater than 4% in Nocodazole).

We observed blebs formed on 5 µM Cytochalasin D treated HeLa and MDCK cells, consistent with previous findings (Meek and Davis, 1986). Nocodazole is known to be a highly reversible drug (Zieve et al., 1980), whereas high concentrations of Cytochalasin D trigger cell death in some cell lines but not in others (White et al., 2001; Kulms et al., 2002; Ailenberg and Silverman, 2003). The morphological changes, including blebbing, in cells treated with 5 µM Cytochalasin D might be interpreted as early signs of apoptosis. To rule out the possibility that the protein density increase in cells treated with Cytochalasin D was caused by apoptosis, we measured mass densities in cells treated with a low concentration of Cytochalasin D at 1 µM. The cells were barely distinguishable from control cells in their morphology, and their trends of mass density changes were consistent with the cells treated with 5 µM Cytochalasin D (Supplementary Figures S8Q–T). Thus, we concluded that the protein density increase in Cytochalasin D was the effect of actin perturbation. Bleb formation indicates a detachment of plasm membrane from cortex and a positive hydrostatic pressure difference (outward pressure) across the plasma membrane (Dai and Sheetz, 1999; Peukes and Betz, 2014). Such outward hydrostatic pressure seems contradictory to the observed cell volume decrease (Supplementary Figures S8B,J). Furthermore, blebbing of Cytochalasin D treated cells seems paradoxical as well. Cortical tension generates the hydrostatic pressure and drives bleb expansion (Tinevez et al., 2009). However, with Cytochalasin D treatment, cortical tension drops dramatically, and blebbing would expect to cease (Tinevez et al., 2009; Peukes and Betz, 2014). What causes the bleb formation and the volume decrease in Cytochalasin D treatment require further investigation.

We further investigated mass density change upon trypsinization which alters cytoskeleton organization in a manner different from the cytoskeletal drugs. Trypsin is a digestive protease normally secreted into the small intestine, that breaks down proteins generally. But in the context used here, it digests the extracellular matrix and extracellular domains of integral plasma membrane proteins, resulting in cell detachment from its proteinaceous substrate. The detachment of the cell from the substrate disrupts actin filament and cortical microtubule organization but does not depolymerize them as does Cytochalasin D or Nocodazole (Furcht and Wendelschafer-Crabb, 1978). After resuspending the trypsin-dissociated cells in the complete medium and plating them in a glass-bottom dish, we monitored their spreading under the NoRI microscope. We found that cells spread slowly on uncoated glass and that the protein density in all three compartments did not change significantly in the first 30 min to 2 h after plating (Supplementary Figures S9). When we compared trypsinized cells 1-h after replating with the untreated cells, we found that trypsinization caused a 7%–12.5% increase in cytoplasmic protein density, an 8%–9% increase in nucleoplasmic protein density, a 1%–8% increase in nucleolar protein density, and a 4%–10% decrease in cytoplasmic lipid density (Figures 5Q–T). Although the effect of trypsinization on cytoskeleton is less dramatic than Cytochalasin D or Nocodazole, we found that it caused more pronounced changes in mass densities. The density changes we observed in trypsinized cells might be a combined effect of cytoskeleton disruption and mechano-osmotic feedback during cell spreading (Venkova et al., 2022). It was surprising to us that lipid changed in the opposite direction from proteins, which would argue that the effects we observed were not just due to changes in volume. Although quantitative regulation of cellular lipid of non-adipocyte cells is not well understood, a recent publication reported a rapid loss of lipid during mitosis (Miettinen et al., 2022), suggesting that exocytosis can have a sizable effect on the lipid mass. An understanding of the nature of lipid mass changes during perturbation of cell shape and cell volume will require future studies.

### Paradoxically protein mass density is resistant to changes in the rates of protein synthesis and protein degradation

Mass density of protein and lipid in various compartments changes very little with cell size in proliferating cells (Figures 2, 3), suggesting that total metabolites are generally proportional to cell dry mass during cell growth (Rollin, Joanny and Sens, 2022). However, this proportionality may not hold when protein synthesis, protein degradation, or global regulators of growth and degradation like mTOR activity are administered. We might, for example, expect that blocking protein synthesis or blocking protein degradation would perturb cell proteostasis in opposite ways. Such perturbations might change cell protein mass and its composition and also affect the state of free amino acid pools (Ennis and Lubin, 1964; Han et al., 2009; Nofal et al., 2017; Santos et al., 2019). A similar expectation would apply to inhibition of mTOR activity, which suppresses protein synthesis and promotes protein degradation (Zhao et al., 2015). mTOR is known to decrease cell dry mass, modulate the production of metabolites, and alter the expression of amino acid transporters (Inoki et al., 2005; Roos et al., 2009; Tucci et al., 2013). We therefore expected dramatic effects of direct pharmacological inhibition of protein synthesis, protein degradation, and mTOR activity on the concentration and composition of impermeant molecules, including protein and small metabolites, and greatly affect mass density. To test these expectations, we quantified mass densities in HeLa, MDCK, and NIH3T3 cells treated with the protein synthesis inhibitor, Cycloheximide, the protein degradation inhibitor, MG132, and the mTOR inhibitor, Rapamycin. As the change of cell dry mass and gene expression profile takes hours to reach the new steady state, we measured the cells after 24 h of treatment with these very well-characterized inhibitors.

**FIGURE 6:**
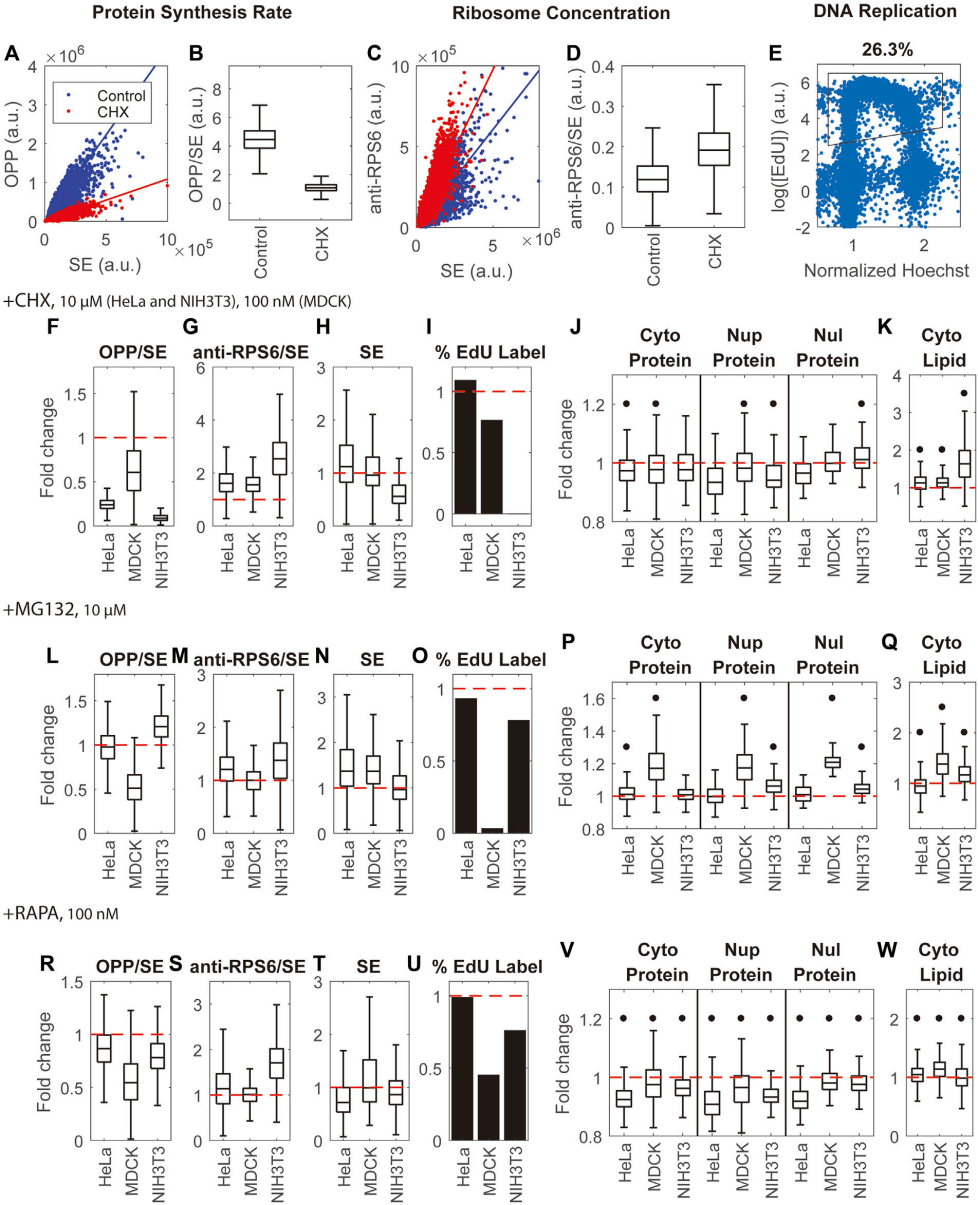
Inhibition of protein synthesis, protein degradation, and mTOR activity have little effect on mass density. **(A,B)** Quantification of protein synthesis rate by the ratio of OPP pulse label to SE protein stain (OPP/SE) demonstrated by untreated HeLa cells and HeLa cells treated by Cycloheximide (CHX). Solid lines in **(A)** are y = kx, where k is the median of the OPP to SE ratio. **(C,D)** Quantification of ribosome concentration by the anti-RPS6 immunostain to SE protein stain ratio (anti-RPS6/SE) demonstrated by untreated HeLa cells and HeLa cells treated by CHX. Solid lines in **(C)** are y = kx, where k is the median of the anti-RPS6 to SE ratio. **(E)** Quantification of DNA replication by EdU labeling in untreated HeLa cells. The X-axis is the nuclear Hoechst intensity normalized by the highest peak of Hoechst distribution. The Y-axis is the logarithm of mean intensity of EdU in the nucleus. Each blue dot is a cell. The black outline is the gate for EdU label cells. **(F–I)** Fold change of protein synthesis rate (OPP/SE) **(F)**, ribosome concentration (anti-RPS6/SE) **(G)**, cell dry mass (SE) **(H)**, and DNA replication (percentage of EdU labeled cells) **(I)** in CHX treated cells **(J,K)** Fold change of protein densities **(J)** and cytoplasmic lipid density **(K)** in CHX treated cells. **(L–O)** Fold change of protein synthesis rate (OPP/SE) **(L)**, ribosome concentration (anti-RPS6/SE) **(M)**, cell dry mass (SE) **(N)**, and DNA replication (percentage of EdU labeled cells) **(O)** in MG132 treated cells. **(P,Q)** Fold change of protein densities **(P)** and cytoplasmic lipid density **(Q)** in MG132 treated cells. **(R–U)** Fold change of protein synthesis rate (OPP/SE) **(R)**, ribosome concentration (anti-RPS6/SE) **(S)**, cell dry mass (SE) **(T)**, and DNA replication (percentage of EdU labeled cells) **(U)** in Rapamycin (RAPA) treated cells. **(V,W)** Fold change of protein density **(V)** and cytoplasmic lipid density **(W)** in Rapamycin treated cells. **(F–W)** are normalized to the median of untreated cells. Red dashed lines in **(F–W)** indicate fold change = 1 (no change). Black dots in **(J–K,P–Q,V–W)** denote significant changes over controls. The absolute changes and representative NoRI images are in Supplementary Figures S10–12. Drug concentrations used in this assay are 100 nM cycloheximide for MDCK cells, 10 µM cycloheximide for HeLa and NIH3T3 cells, and 10 µM MG132 and 100 nM Rapamycin for all 3 cell lines.

Different cell lines are known to have different drug sensitivities and different physiological responses; therefore, we examined the effects of these inhibitors on 3 cell lines. We monitored the effects of these treatments on protein synthesis rates by the ratio of pulse-labeled OPP (O-propargyl-puromycin) to the SE protein stain, which would monitor the rate of protein synthesis per unit protein mass (Figures 6A,B). As expected, Cycloheximide and Rapamycin inhibited protein synthesis in different cell lines by 0.09–0.61 and 0.54–0.87 fold, respectively, when delivered at the same dose (Figures 6F,R). The effect of the proteasome inhibitor MG132 was more variable. It caused a 0.51-fold decrease in protein synthesis rate in MDCK cells, a 1.21-fold increase in NIH3T3 cells, and no change in HeLa cells (Figure 6L). These inhibitors also had clear effects on cell dry mass (Figures 6H,N,T). The most pronounced changes were a 1.37 fold increase in dry mass in HeLa and NIH3T3 cells treated with MG132, a 0.56 fold decrease in dry mass in NIH3T3 cells treated with Cycloheximide, and a 0.72 fold decrease in dry mass in HeLa cells treated with Rapamycin. The effect on the cell cycle of these protein synthesis and degradation inhibitors also varied. By quantifying the percentage of EdU-labeled cells (Figure 6E), we could track replication of DNA. We found that most of the drugs and cell line combinations had little effect or simply slowed down the cell cycle (Figure 6I,O,U). The two exceptions were NIH3T3 cells treated with Cycloheximide and MDCK cells treated with MG132, where the cell cycle was arrested almost completely. Since the ribosome comprises a sizable fraction (around 6%) of the protein content in mammalian cells (An et al., 2020), we investigated whether the ribosome concentration (quantified by the ratio of anti-RPS6 immunostain to the SE protein stain) correlated with mass density. Although ribosome subunit proteins are commonly used to quantify ribosome content, this has limitations because certain conditions may generate more unassembled ribosome proteins and break the proportionality between ribosome proteins and assembled ribosomes (Zhang et al., 2016; An and Harper, 2020). Thus the anti-RPS6/SE measurements should be interpreted with caution. Nevertheless, we found that the three inhibitors either had negligible effects on or increased ribosome concentration (Figures 6G,M,S). The pronounced changes in anti-RPS6 to SE ratio were a 2.6-fold increase in NIH3T3 cells in Cycloheximide, a 1.7-fold increase in NIH3T3 cells in Rapamycin, and a 1.6-fold increase in HeLa and MDCK cells in Cycloheximide.

The general conclusion from all the pharmacological treatments on multiple cell lines is that most drug treatments on the various cell lines had unexpectedly small effects on *mass density*, despite their dramatic effects on protein synthesis and degradation. There were two exceptions: NIH3T3 cells in Cycloheximide and MDCK cells in MG132, where in both cases the cell cycle was arrested. Several measurements of pharmacologic effects on mass density, though small, were statistically significant since they involved a very large number of cells (150–1000). To compare the effects of the drugs on different cell lines, we plotted the inhibitors’ effects on mass density as relative changes. We found that Rapamycin decreased protein density in all measured compartments of all cell lines by 2%–9% and altered lipid density by 1%–14% (Figures 6V,W). The effect of Cycloheximide was smaller. It decreased protein density by 0%–7% and increased lipid density by 13% in HeLa and MDCK cells (Figures 6J,K). MG132 increased protein density by 1–7% and altered lipid density by 7%–16% in HeLa and NIH3T3 cells (Figure 6). The relative changes were either smaller than or comparable to the spontaneous cell-to-cell variability of these cell lines. The outlier cases, which suffered arrest of the cell cycle, showed larger effects on mass density: NIH3T3 cells in Cycloheximide showed a 2%–6% change in protein densities and a 63% increase in lipid density; MDCK cells in MG132 showed a 17%–21% increase in protein densities and a 38% increase in lipid density.

### Cell senescence and starvation dramatically change cell mass density

We demonstrated that cellular protein mass density for a given cell type is stable over multiple passages, stable throughout the cell cycle, and robust to perturbation by drugs that dramatically affect cell protein mass, such as inhibitors of protein synthesis and degradation. The resistance of mass density to perturbation suggests that there is some kind of feedback that effectively maintains the protein mass density against diverse perturbations. To begin to define this feedback mechanism, we looked for perturbations that significantly affect mass density either by overwhelming the purported feedback or acting through some other regulatory pathways. The two we considered are senescence, caused by DNA damage, and quiescence, induced by serum starvation.

**FIGURE 7:**
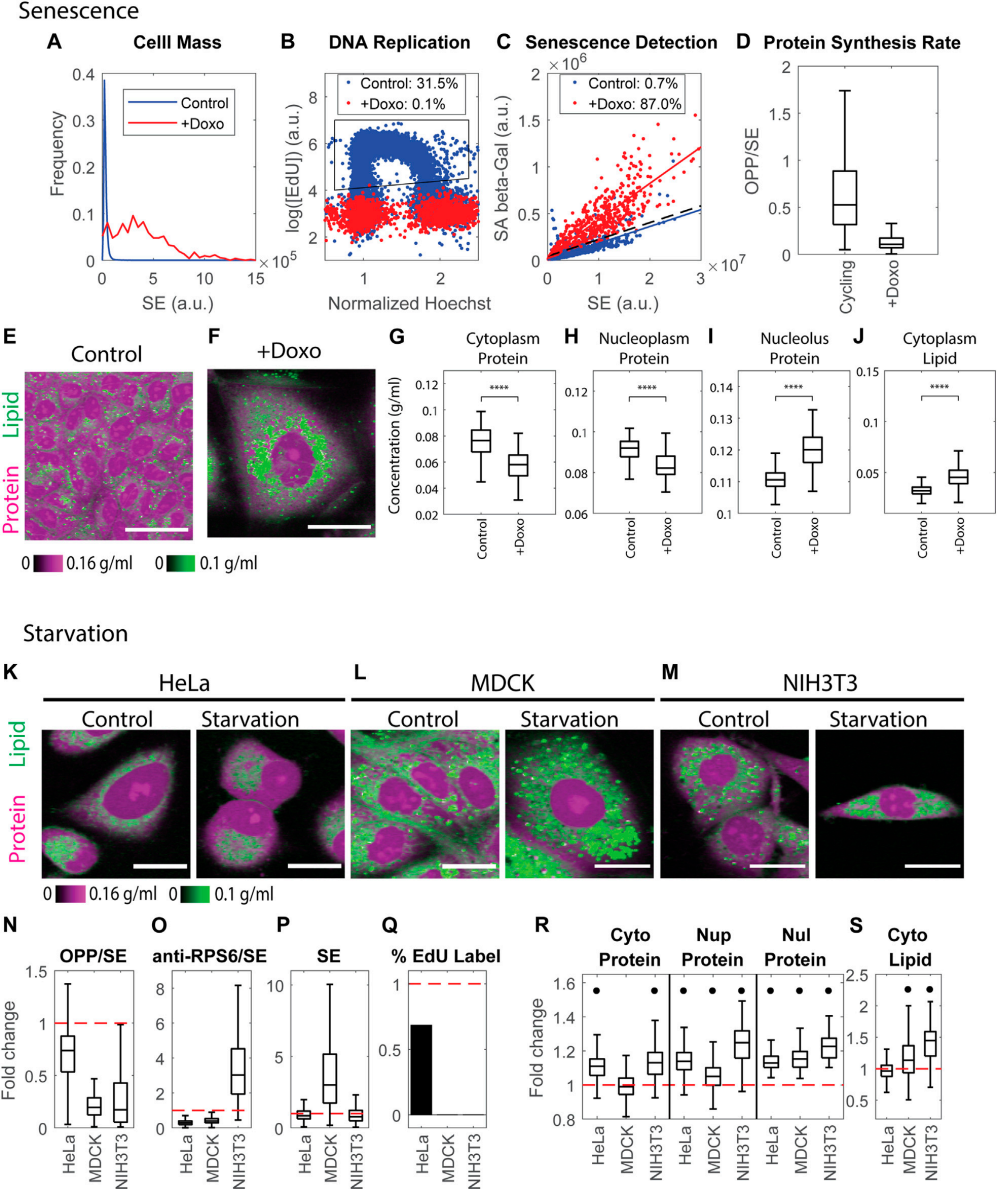
Cell senescence and quiescence change mass densities dramatically. **(A)** Cell mass distributions quantified by the SE protein stain in untreated MDCK cells and MDCK cells treated with 100 ng/ml Doxorubicin for 5 days. *n* = 26391(Control), *n* = 523 (+Doxo). **(B)** DNA replication detected by EdU incorporation in untreated MDCK cells and MDCK cells treated with 100 ng/ml Doxorubicin for 5 days. *n* = 17816 (Control), *n* = 1939 (+Doxo). Legends indicate the percentage of cells in the gated region (black polygon) of EdU incorporation. **(C)** Senescent cells detected by SA-β-galactosidase activity vs. SE protein stain. Each dot is an individual cell. Solid colored lines are the linear fit of the correlation. The dashed black line y = ax + 3b is the threshold used to detect senescent cells, where a is the slope of the linear fit of the control correlation, and b is the Root Mean Square Error (RMSE) of the fit. Legends indicate the percentage of senescent cells detected by this threshold. **(D)** Protein synthesis rate quantified by the OPP/SE ratio in cycling cells (Control *n* = 20481), and senescent cells (+Doxo *n* = 1134). **(E,F)** Protein and lipid concentration image of cycling **(E)** and senescent **(F)** MDCK cells. Scale bar is 40 µm. **(G–J)** Cytoplasmic protein density **(G)**, nucleoplasm protein density **(H)**, nucleolus protein density **(I)**, and cytoplasm lipid density **(J)** in senescent MDCK cells (+Doxo *n* = 112), compared to cycling cells (Control *n* = 1169). **(K–M)** Representative NoRI images of control and 0.1% serum-starved HeLa **(K)**, MDCK **(L)**, and NIH3T3 **(M)** cells. Scale bar, 20 µm. **(N–Q)** Fold change of protein synthesis rate quantified by the ratio of pulse-labeled OPP to SE protein stain (OPP/SE) **(N)**, ribosome concentration quantified by the ratio of anti-RPS6 immunostain to SE protein stain (anti-RPS6/SE) **(O)**, cell dry mass (SE) **(P)**, and DNA replication (percentage of EdU labeled cells) **(Q)** in serum starved cells normalized to the median of control cells. Red dashed line indicates change fold = 1 (no change). **(R,S)** Fold change of protein densities and cytoplasmic lipid density in serum starved cells normalized to the median of control cells. Red dashed lines indicate change fold = 1 (no change). Black dots denote significant changes. The absolute changes in **(N–S)** are summarized in Supplementary Figures S13.

A common way to induce cellular senescence is by inhibiting the cell cycle through DNA damage. We treated MDCK cells with the genotoxic drug, Doxorubicin, for 5 days. As expected, cell dry mass had increased massively at the endpoint of the treatment. Measurement with the SE protein dye revealed an average 12.6-fold increase in cell dry mass (Figure 7A). Furthermore, most cells had exited the cell cycle either before or after DNA replication, as indicated by the negligible level of EdU incorporation (0.1%) (Figure 7B). We further confirmed the senescence phenotype by the high level of senescence-associated β-galactosidase (SA-β-galactosidase) activity (Dimri et al., 1995) in the treated cells, correcting for the increase expected from the increased cell mass (Lee et al., 2006) (Figure 7C). Using this criterion, we found that 87% of treated cells were senescent. The protein synthesis rate in the senescent cells was drastically reduced, as the OPP to SE ratio was only one-fifth of that of control cells (Figure 7D). Thus, we concluded the vast majority of treated cells had reached a state of senescence.

In the starvation experiments, we confirmed the quiescent phenotype by the documented reduced protein synthesis and cell cycle arrest. MDCK and NIH3T3 cells starved in 0.1% serum for 5 days were completely arrested in the cell cycle; in addition, the rate of protein synthesis decreased by more than 80% (Figures 7N,Q). However, HeLa cells (perhaps reflecting their origin as an aggressive tumor) were more resistant to serum starvation. After being cultured in 0.1% serum for 5 days, the cell cycle was only partially inhibited, and protein synthesis decreased by only 25% (Figures 7N,Q). We concluded that most of the serum-starved MDCK and NIH3T3 cells had reached some form of quiescent state, but HeLa cells might not have achieved what is considered the typical quiescent state. Due to the reduced rates of protein synthesis, we might have expected that starvation-induced quiescence would be accompanied by lower expression in RPS6 and have resulted in reduced cell mass (Alessio et al., 2021). However, we found the phenotypes were inconsistent among cell lines. HeLa and MDCK cells had a lower RPS6 concentration (0.29 or 0.4 fold of the control cells, respectively), whereas NIH3T3 cells increased RPS6 concentration by three fold (Figure 7O). The dry mass of HeLa and NIH3T3 was 0.85 or 0.78 fold of the control cells, whereas serum-starved MDCK cells increased their dry mass by three fold (Figure 7P).

Senescence and quiescence share similarities in cell cycle repression, elevated SA-β-galactosidase activity, increase in lysosome content, and the changes in their transcriptomic profiles (Cho and Hwang, 2012; Fujimaki et al., 2019; Alessio et al., 2021). Cells in quiescence that have been starved for many days can gradually shift into a senescence-like state by increasing lysosome biogenesis and decreasing autophagy (Fujimaki et al., 2019). In stark contrast to the results of protein synthesis inhibition presented in the previous section, both senescent and quiescent states were dramatically altered in their protein and lipid *mass densities*. Furthermore, despite some similarities between senescence and quiescence, the resultant mass densities were altered in different directions. Consistent with previous findings (Okumura et al., 2015; Flor et al., 2017; Neurohr et al., 2019; Oh et al., 2022), we found that senescent cells had a significantly diluted cytoplasmic protein density (0.87 fold compared to control cells) (Figure 7G). The nucleoplasm protein density was diluted to a similar extent (0.9 fold) (Figure 7H). Surprisingly, nucleolus protein density increased by 1.08 fold (Figure 7I), and cytoplasmic lipid density also increased greatly by 1.66 fold (Figure 7J), which was consistent with previous findings (Flor et al., 2017; Oh et al., 2022).

In contrast to senescent cells, quiescent cells *increased* cytoplasm and nucleoplasm protein density (Figure 7R). Starved HeLa and NIH3T3 cells increased cytoplasmic protein density by 1.11–1.13 fold. By contrast, the change in cytoplasmic protein density in MDCK cells was negligible (Figure 7R). Nucleoplasm protein density increased by 13%, 5%, and 25% in HeLa, MDCK, and NIH3T3 cells, respectively (Figure 7R). The nucleolus protein density also increased by 13%–23% in all 3 cell lines (Figure 7R). Cytoplasm lipid density increased in MDCK cells by 1.13 fold and in NIH3T3 cells by 1.45 fold, yet the change in HeLa cells was negligible (Figure 7S). The effects of starvation on mass densities were generally more pronounced than the effects of Rapamycin. Although starvation causes mTOR inhibition, its impact on cell physiology must encompass a wider range of targets.

## Discussion

Cell size is itself an ambiguous term. In various contexts it might refer to a cell’s longest dimension, its area, volume, total mass, dry or buoyant mass, protein, lipid, or carbohydrate content and other macromolecular parameters. When the structure and composition of the cell over time remains uniform, these parameters should vary in parallel, meaning that each can equally serve as a measure of growth. But when they do not vary in parallel, then we have to ask what we mean by growth and ask how each might be independently regulated. Until recently it has been very difficult to characterize growth accurately enough to answer such a question. Now with improved methods, we can gain insight into the regulation of volume, mass, and mass density and can characterize their interdependencies. Although there have been efforts to measure protein mass, lipid mass has seldom been evaluated. Previous measurements have shown that total cell dry mass is mostly regulated through protein synthesis and degradation and is very tightly controlled throughout the cell cycle to maintain cell mass homeostasis (Liu, Yan and Kirschner, 2022). By contrast, cell volume is known to vary with changes in the external milieu, responding rapidly to mechanical and osmotic stress (Hoffmann, Lambert and Pedersen, 2009; Xie, Yang and Jiang, 2018; Wang et al., 2021). Furthermore, even in the absence of such stress, cell volume swells during the prophase and prometaphase (Son et al., 2015; Zlotek-Zlotkiewicz et al., 2015) by more than 10% and fluctuates with cell shape changes, such as cell spreading (causing several percent loss in cell volume) (Venkova et al., 2022). These studies give the impression that cell dry mass and volume are independently regulated or only loosely coupled (Neurohr and Amon, 2020). However, if mass and volume were independently varying, we would expect to also see mass density (mass/volume) reflect this. Mass density changes should be consequential, since volume changes would affect all reactions, whose rate would be determined by the concentration of reactants. The effect of concentration change might be especially pronounced in highly cooperative transitions that operate near the switching threshold, such as those occurring in protein complexes (Neurohr and Amon, 2020). Reactions of high degrees of cooperativity or order could show higher sensitivity to reactant concentration, where even a small change would result in substantial changes in reaction rates or equilibrium state and perturb certain pathways (Ferrell and Ha, 2014). For this reason, we would anticipate that the cell might need to sensitively control mass density. The development of a new quantitative imaging modality for proteins and lipids, called Normalized Raman Imaging (NoRI), now allows us to address these questions through precise and direct measures of protein and lipid mass density.

Is a given mass density simply a resultant of all the reactions, synthetic and degradatory, in the cell? How fragile is that balance? We tested the resilience of cell mass density by treating cells pharmacologically with protein synthesis inhibitors, inhibitors of the proteasome, or broad inhibitors of protein metabolism by the mTOR inhibitor, Rapamycin. Such inhibitors would be expected to change the proteome and the metabolic networks significantly. As expected, these drugs strongly affect protein synthesis and degradation rates, ribosome concentration, and total cell dry mass (Figure 6), but to our surprise, they leave protein mass density essentially unchanged. This resilience suggests some form of homeostatic regulation of the mass density steady state.

Though protein mass density is remarkably stable in proliferating cells, it is not unalterable. We observed apparently spontaneous variation in mass density in unperturbed MDCK cells. Notably, this variation was strongly correlated with YAP activity in the cells (Figures 2K–N). It has already been shown that cell volume responds to mechanical stimuli and substrate rigidity through the YAP/TAZ dependent pathway; it has not previously been known how it affects mass density (Dasgupta and McCollum, 2019; Pocaterra, Romani and Dupont, 2020). Mass density also changes in global transitions of cell states, such as differentiation and in senescence (Cooper et al., 2013; Okumura et al., 2015; Neurohr et al., 2019; Oh et al., 2022). We have also observed mass density changes when cells enter a different physiological state, such as cell cycle arrest caused by the protein synthesis inhibitor Cycloheximide in NIH3T3 or cell cycle arrest caused by the proteasome inhibitor MG132 in MDCK cells (Figure 6), by serum starvation, and by genotoxic drug-induced senescence (Figure 7). In these circumstances, the perturbations must be able to circumvent whatever homeostatic mechanisms maintaining mass density. We surmise that global cell state changes like cell differentiation can override mass density control and probably reflect discrete changes in the proteome. The tradeoff between maintaining mass density and altering it may ultimately be significant in optimal cell function and may also fail in disease.

Since NoRI is a high-resolution microscopic technique, we have the ability to examine the protein density of subcellular compartments. Nuclear protein density is regulated very similarly to cytoplasmic protein density, but nucleolar protein density is not. We find a very consistent ratio of protein densities in the nucleolus, nucleoplasm, and cytoplasm, of 1.5:1.1:1, in 3 cell lines representing different cell types (Figure 2J). Particularly, the ratio of protein densities in the nucleoplasm and cytoplasm were essentially independent of the osmolarity of the extracellular medium, conditions where cytoplasmic protein density changes over a large range from 0.055 to 0.105 g/ml (Figure 4I). When corrected for the contribution of nucleic acid to the observed nucleoplasmic protein density (about 10%), the ratio indicates that the nucleoplasmic protein density is very close to the cytoplasmic protein density; both change in parallel. The nuclear envelope is known to be permeable to ions, metabolites, and small proteins (Mazzanti, Bustamante and Oberleithner, 2001) but only semi-permeable to larger proteins (larger than 60 kD) (Silver, 1991) and to RNA and, of course, impermeable to chromatin. The changes in osmotic pressure across the nuclear envelope are presumably determined by imbalances caused by active protein transport in and out of the nuclear compartment and less by the osmolarity of the impermeable chromatin and its associated ions (Deviri and Safran, 2022; Lemière et al., 2022). The equal protein densities on both sides of the nuclear envelope demonstrate that, like the plasma membrane, membrane tension on the nuclear envelope is very small. Consistent with the models proposed by Deviri and Safran (2022); Lemière et al. (2022), the ratio between nuclear and cytoplasmic volumes is proportional to the ratio between the number (not mass) of the nuclear and cytoplasmic proteins. Thus, this proportionality should be independent of pressure and tension on the plasma membrane (Deviri and Safran, 2022; Lemière et al., 2022). This proportionality, determined by the colloidal osmotic pressure inside and outside the nuclear envelope, could serve as the mechanism for maintaining a stable nuclear-cytoplasm (N/C) ratio, as seen in many studies (Trombetta, 1942; Jorgensen et al., 2007; Neumann and Nurse, 2007; Tsichlaki and FitzHarris, 2016). To confirm this conjecture, further studies of nucleoplasmic and cytoplasmic protein densities should be done in other cell types; The 4-band NoRI (Oh et al., 2022), which independently measures protein, lipid, nucleic acid, and water density would be ideal for such studies.

Regulation of lipid density appears quite distinct from regulation of protein density. NoRI separately measures lipid mass density and its spatial distribution relative to protein. We find that lipid mass density is less constrained than protein mass density, with much larger variation in cells of the same cell type. For example, in A7 melanoma cells, the CV of lipid concentrations is 30%, while the CV for protein is 7% (Figure 1J). The CV of cytoplasmic lipid density in live HeLa, NIH3T3, and MDCK cells lies in the range of 14%–17%, while the CV for cytoplasmic protein density is 5%–11% (Figure 2I). Perhaps the lipid distribution should be considered more in terms of a phase separated domain than a solute. Indeed, compared to protein density, lipid density indicated a much larger dispersion in single cross-section vs. whole cell body correlation and a larger pixel-to-pixel variation within a cell (Supplementary Figures S1). Nevertheless, there is a positive correlation between lipid density within a cell type with changes in protein mass density under osmotic stress or cytoskeletal disruption (Figures 3, 4), showing that lipid density may also be affected by cell volume. However, this correlation is inverted when cells are treated with Cycloheximide or Rapamycin (Figure 6). Furthermore, the amplitude of lipid density changes is less consistent among different cell types than the amplitude of protein density changes. The lipid density responses to protein synthesis and mTOR inhibitors are most likely due to the very large differences in the regulation of lipid metabolism and protein with these drugs (Arbogast and Henderson, 1975; García-Sáinz, Piña and Chagoya de Sánchez, 1977; Roberts et al., 1982; Soliman, 2011; Ricoult and Manning, 2013). On the other hand, the lipid distribution can be very sensitive to perturbation in some cell types and may reflect redistribution between the Golgi-ER system and the plasma membrane. Large lipid density increases in cells other than adipocytes are known markers of physiological states, such as senescence, apoptosis, neuron damage, intracellular pathogens, and cancer (Flor et al., 2017; Shyu et al., 2018; Geltinger et al., 2020). Therefore, changes in lipid distribution may be of particular physiological significance and deserve increased attention.

To maintain homeostasis, a cell must have some way to sense protein mass density. Unlike total (dry) mass and total volume, which could in principle be sensed by processes of titration (Amodeo and Skotheim, 2015) or, more generally, by subscaled inhibitors and superscaled activators (Chen et al., 2020; Lanz et al., 2021; Xie, Swaffer and Skotheim, 2022), cells seem to be optimizing and maintaining a constant mass density against fluctuation and perturbation, while at the same time being insensitive to the total dry mass. Perhaps to do this, they utilize reactions that are ultrasensitive to density changes. It is known that the MAPK pathway is the major pathway in mammalian cells that responds to osmotic stress (de Nadal, Alepuz and Posas, 2002; Zhou et al., 2016). It is thought to modulate cell volume, metabolite composition, protein synthesis, and protein degradation (Cargnello and Roux, 2011). Perhaps it may also regulate density. How exactly mass density is sensed, what are the signaling pathways that connect it to the massive biosynthetic control and volume regulation, and how downstream effects of the pathways might compensate for stochastic perturbation are currently the key unanswered questions.

In summary, the uniformity and stability of protein mass density suggests that it may itself be the target of stringent regulation. The variation of mass density in different cell types and in different physiological states suggests it has an important role in cell physiology. How this would work is still completely unknown. For future studies, NoRI, with its vastly improved quality and ease of direct measurement of protein density and its ability to separately measure protein and lipid, would seem to be an important tool for directly reporting on mass density in diverse experimental settings, including living or fixed cultured cells or tissues. Ultimately, we will need to tie the quantitative measurement of mass density to biological circuits that regulate cell volume, metabolism, protein expression, and protein modification and study these in diverse circumstances of differentiation, cell cycle, and pathology.

## Data Availability

The raw data supporting the conclusion of this article will be made available by the authors, without undue reservation. The code used in this work is available at https://github.com/kirschnerlab/NoRI.
