# Supplementary material for: The uniformity and stability of cellular mass density in mammalian cell culture
Source: Front Cell Dev Biol. 2022 Oct 12;10:1017499. doi: 10.3389/fcell.2022.1017499 (PMC9597509; doi:10.3389/fcell.2022.1017499)
Supplement: Supplementary file 1 [file Image1.pdf]

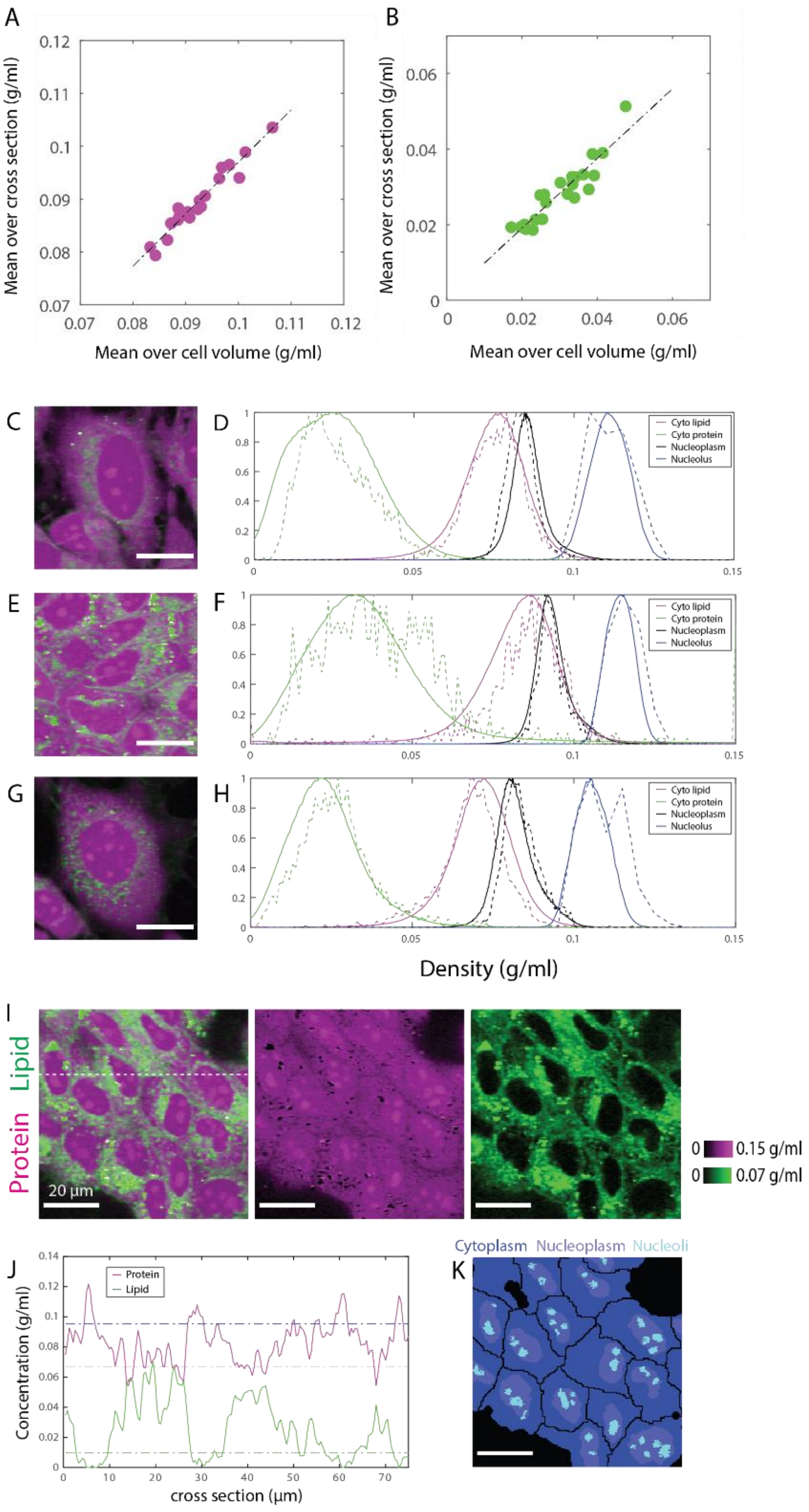

Supplementary Figure S1. The homogeneity of protein and lipid mass density. (A-B) Protein (A) and lipid (B) mass density measured by the average over the whole cell volume compared to the average

in a single z-plane at the mid-cell height. Protein slope=0.98, intercept =0.001, R-squared=0.98. Lipid slope=0.92, intercept =0.001, R-squared=0.85. (C-E) Pixel-by-pixel variability of protein and lipid densities in the cytoplasm (magenta, green), nucleoplasm protein density (black), and nucleolus protein density (blue). Solid lines are the pixel value distribution of all cells pooled together by shifting each cell's mean protein or lipid density to the population's average. Dashed lines show pixel value distribution from a representative single cell shown on the left. Scale bars, 20 µm. (I) Representative NoRI images of live MDCK cells at high confluence. Scale bar, 20 µm. (J) Protein and lipid density profiles along the dashed cross line in (I). The dashed lines indicate the protein (magenta) and lipid (green) thresholds used in the segmentation. (M) Segmentation of (I) indicating cell bodies, nuclei, and nucleoli.

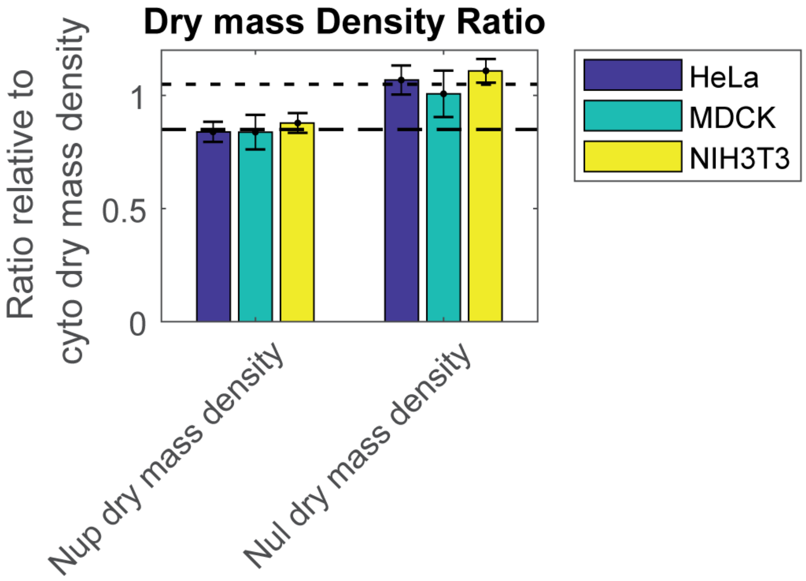

Supplementary Figure S2. The ratio of nucleoplasm or nucleolus dry mass density to cytoplasm dry mass density is constant in the three cell lines investigated. The long dashed line indicates the averaged nucleoplasm to cytoplasm dry mass density ratio of the three cell lines at 0.85. The short dashed line indicates the averaged nucleolus to cytoplasm dry mass density ratio of the three cell lines at 1.05. Nup, nucleoplasm; Nul, nucleolus; Cyto, cytoplasm.

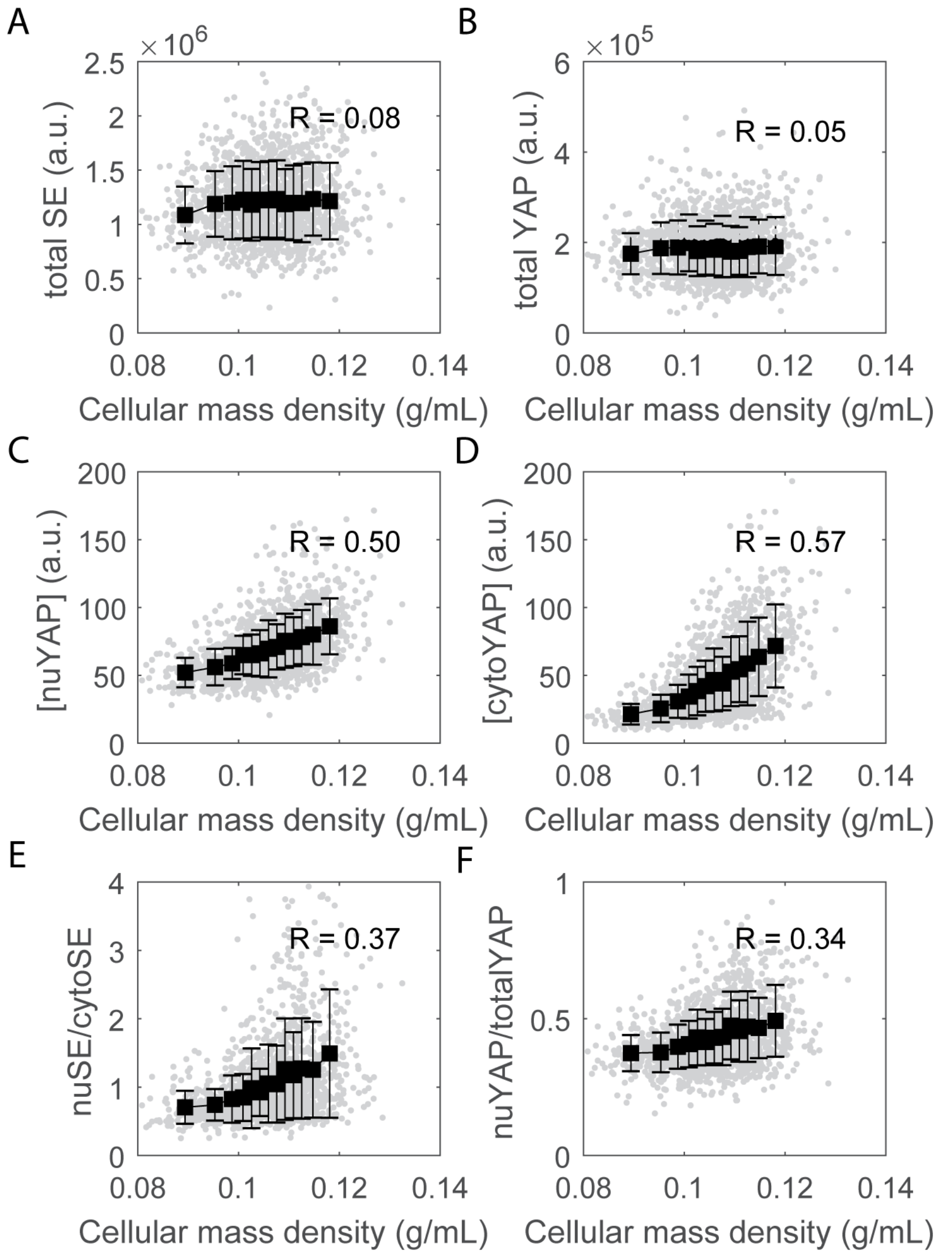

Supplementary Figure S3. Addition correlations between cellular mass density and YAP or cell mass qualities. (A) Cell dry mass is uncorrelated with cellular mass density. (B) Total YAP immunostain is uncorrelated with cellular mass density. (C-D) The mean intensity of nuclear YAP (C), the mean intensity of cytoplasmic YAP (D), the ratio of nuclear to cytoplasmic SE stain (E), and the ratio of nuclear to total YAP immunostain (F), each positively correlate with cellular mass density, . Each grey dot represents a cell; black filled squares are the mean of each bin; error bars indicate the standard deviation of the bin; R is Pearson’s correlation.

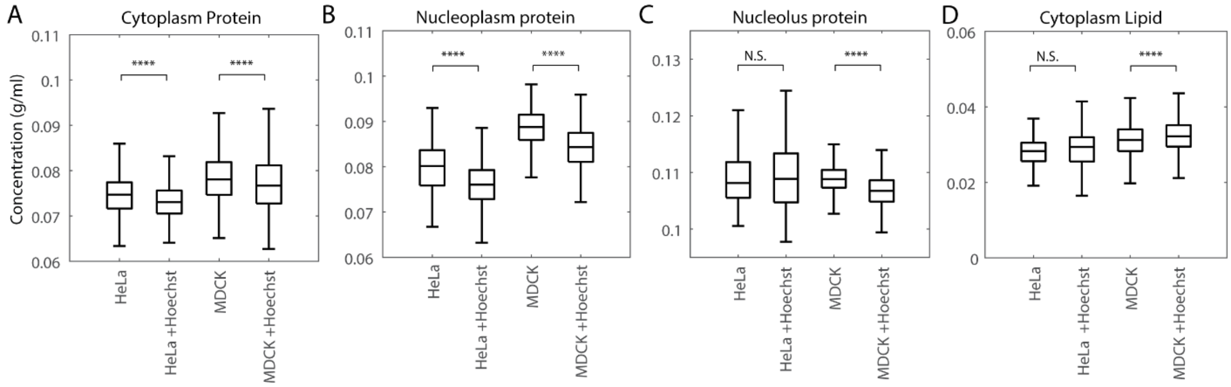

Supplementary Figure S4. The effect of Hoechst stain on mass densities. (A-D) Comparison of mass densities between unstained HeLa or MDCK cells and cells stained with Hoechst. N = 282 (Hela unstained), 418 (HeLa stained), 1204 (MDCK unstained), 1291 (MDCK stained).

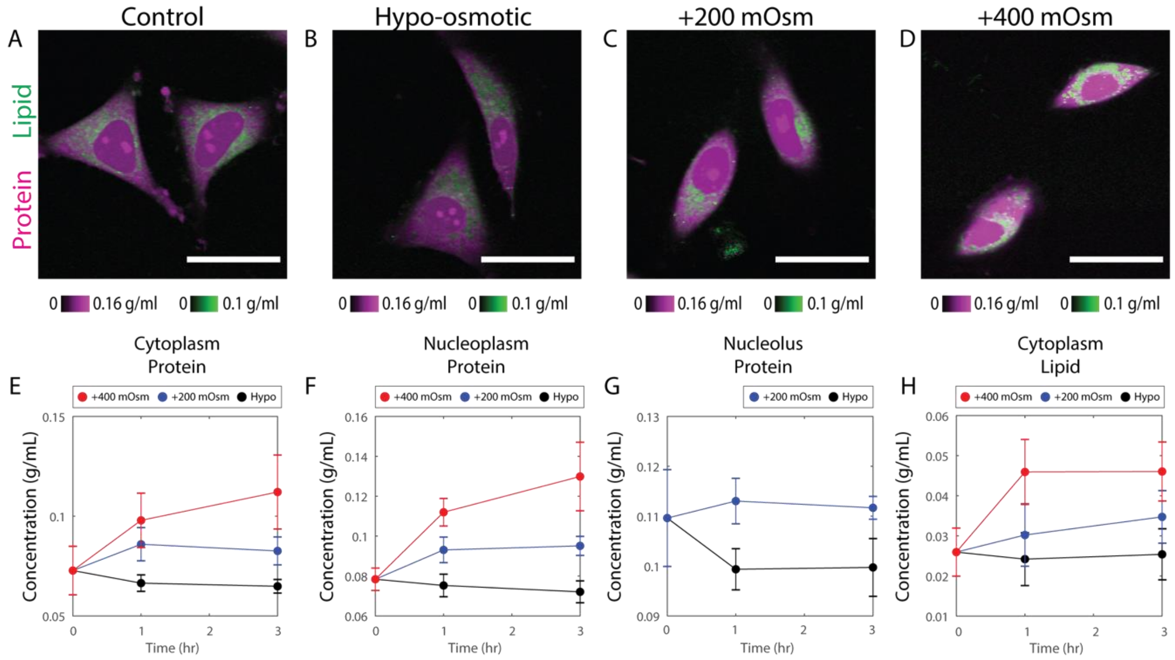

Supplementary Figure S5. Osmotic perturbation of mass density in HeLa cells. (A-D) Representative NoRI images of control HeLa cells in complete medium and HeLa cells after 3 hours in hypo-osmotic or hyper-osmotic media. Scale bar, 20 µm. (E-F) Time course of protein and lipid density change by hyper-osmotic and hypo-osmotic treatment. Time 0 shows the control sample. Data points and error bars are the mean and standard deviation. The number of cells in each data point is between 74 to 263 cells, with a mean of 118 cells.

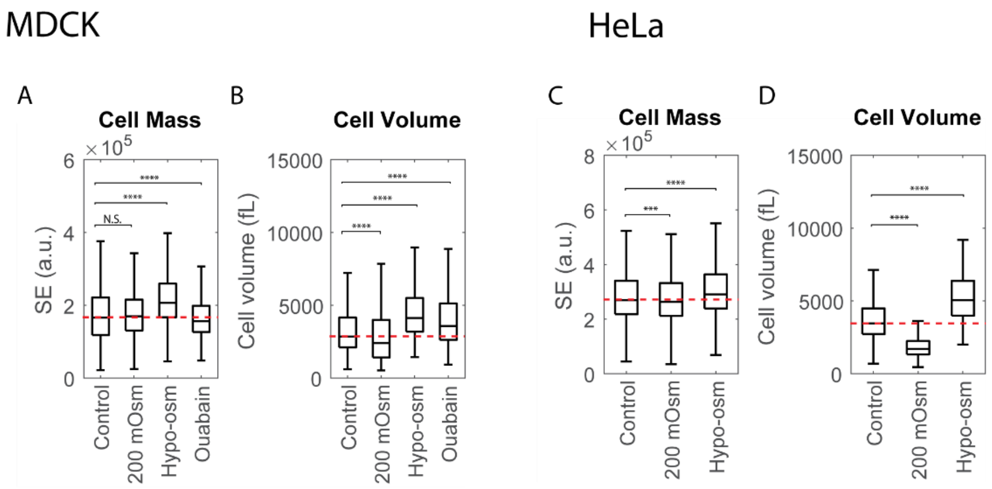

Supplementary Figure S6. Cell size changes with osmotic perturbations in MDCK and HeLa cells. (A, C) Dry mass change estimated by the SE protein stain with osmotic perturbations in MDCK (A) and HeLa (C) cells. (B, D) Cell volume measured by the Coulter principle with osmotic perturbations in MDCK (B) or HeLa (D) cells. Cells were measured after 1-hour treatment in the hypo- or +200 mOsm hyper-osmotic medium or 5-hour treatment in 3 µM ouabain. N=8524-16257 cells (dry mass), N=3145-8000 cells (volume). Red dashed lines in (A-D) indicate the median of the control samples.

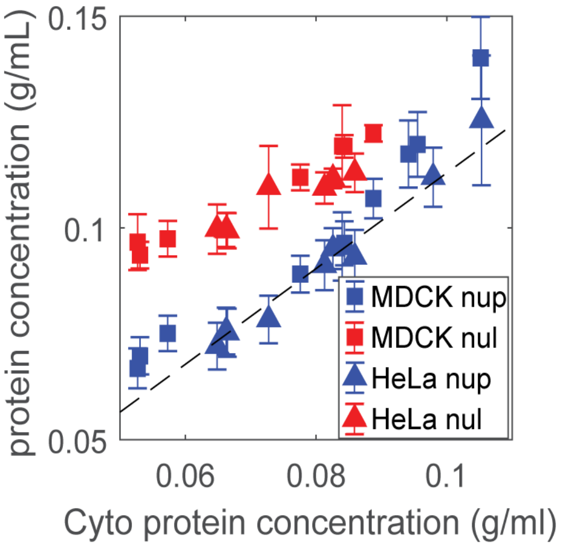

Supplementary Figure S7. Absolute changes in nucleoplasm and nucleolus protein densities with cytoplasm protein density in iso-, hypo-, and hyper-osmotic media. Dashed line, $y = k \cdot x$, where k is the mean ratio between nucleoplasm and cytoplasm protein density in Figure 2J. Nup, nucleoplasm; nul, nucleolus.

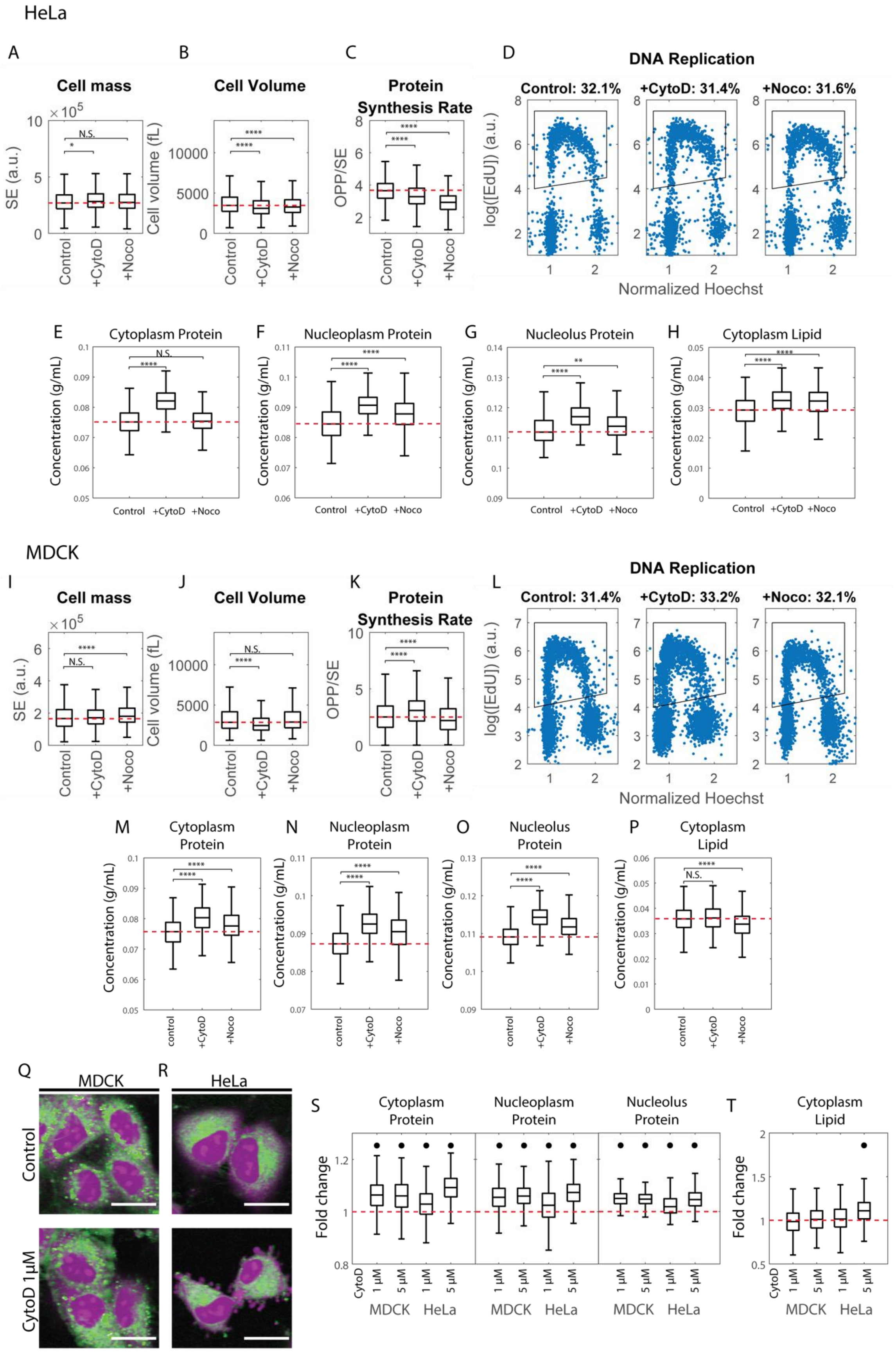
HeLa
A
Cell mass
SE (a.u.)
N.S.
*
Control
+CytoD
+Noco
B
Cell Volume
Cell volume (fL)
****
C
Protein Synthesis Rate
OPP/SE
D
DNA Replication
Control: 32.1%
+CytoD: 31.4%
+Noco: 31.6%
log([EdU]) (a.u.)
Normalized Hoechst
E
Cytoplasm Protein
F
Nucleoplasm Protein
G
Nucleolus Protein
H
Cytoplasm Lipid
Concentration (g/mL)
**
MDCK
I
Cell mass
J
Cell Volume
K
Protein Synthesis Rate
L
DNA Replication
Control: 31.4%
+CytoD: 33.2%
+Noco: 32.1%
M
Cytoplasm Protein
N
Nucleoplasm Protein
O
Nucleolus Protein
P
Cytoplasm Lipid
control
Q
MDCK
R
HeLa
Control
CytoD 1µM
S
Cytoplasm Protein
Nucleoplasm Protein
Nucleolus Protein
Fold change
CytoD
1 µM
5 µM
MDCK
HeLa
T
Cytoplasm Lipid

Supplementary Figure S8. Cytoskeleton perturbation increases protein density in HeLa and MDCK cells. Cells were treated in 5 µM Cytochalasin D (+CytoD) or 5 µM Nocodazole (+Noco) for 1 hour. (A, I) Cell dry mass measured by SE fluorescence. (B, J) Cell volume measured by the Coulter principle. (C, K) Protein synthesis rate quantified by the OPP pulse label to SE protein stain ratio (OPP/SE). (D, L) DNA replication quantified by the percentage of EdU labeled cells. The X axis is the nuclear Hoechst intensity normalized by the highest peak of Hoechst distribution. The Y axis is the logarithm of mean intensity of EdU in the nucleus. Each blue dot is a cell. The black outlines are the gates for EdU labeled cells. The titles indicate the percentage of labeled cells in the whole population. (E-H, M-P) NoRI measurements of protein density in cytoplasm, nucleoplasm, nucleolus, and lipid density in cytoplasm in HeLa cells (N=677 (Control); N=635 (+CytoD); N=313 (+Noco)), and MDCK cells (N=677 (Control); N=550 (+CytoD); N=760 (+Noco)). Red dashed lines indicate the medians of controls. (Q, R) Representative NoRI images of untreated HeLa and MDCK cells and cells treated with 1 µM Cytochalasin D for 1 hour. Scale bar, 20 µm. (S, T) Fold change of protein densities and cytoplasmic lipid density in MDCK and HeLa cells treated with 1 µM or 5 µM Cytochalasin D normalized to the median of untreated cells. Red dashed lines indicate fold change=1 (no change). Black dots denote significant changes compared to control.

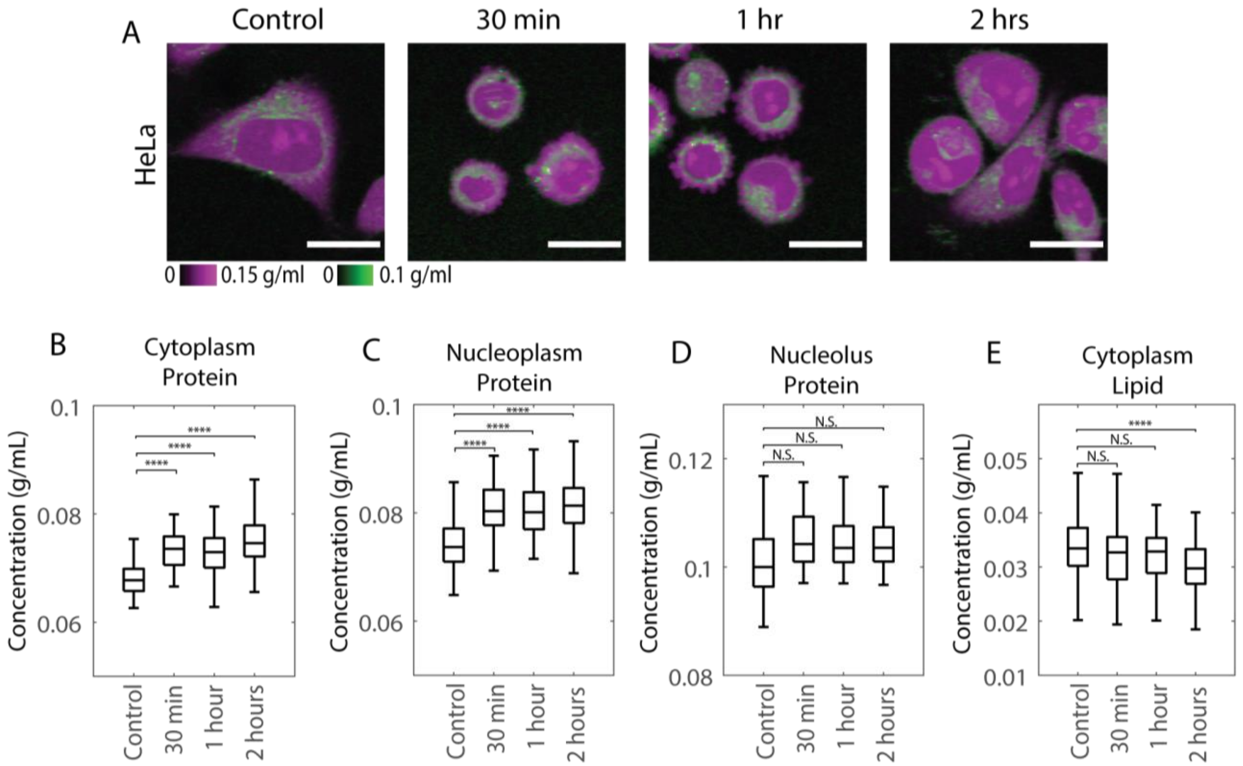

Supplementary Figure S9. Time course of HeLa cell spreading. (A) Representative NoRI images of HeLa cells before trypsinization and at 30 minutes, 1 hour, and 2 hours after plating. (B-E) Cytoplasmic protein density (B), nucleoplasm protein density (C), nucleolus protein density (D), and cytoplasm lipid density (E) in HeLa cells before trypsinization (N=213) and at 30 minutes (N=45), 1 hour (N=108), and 2 hours (N=124) after plating.

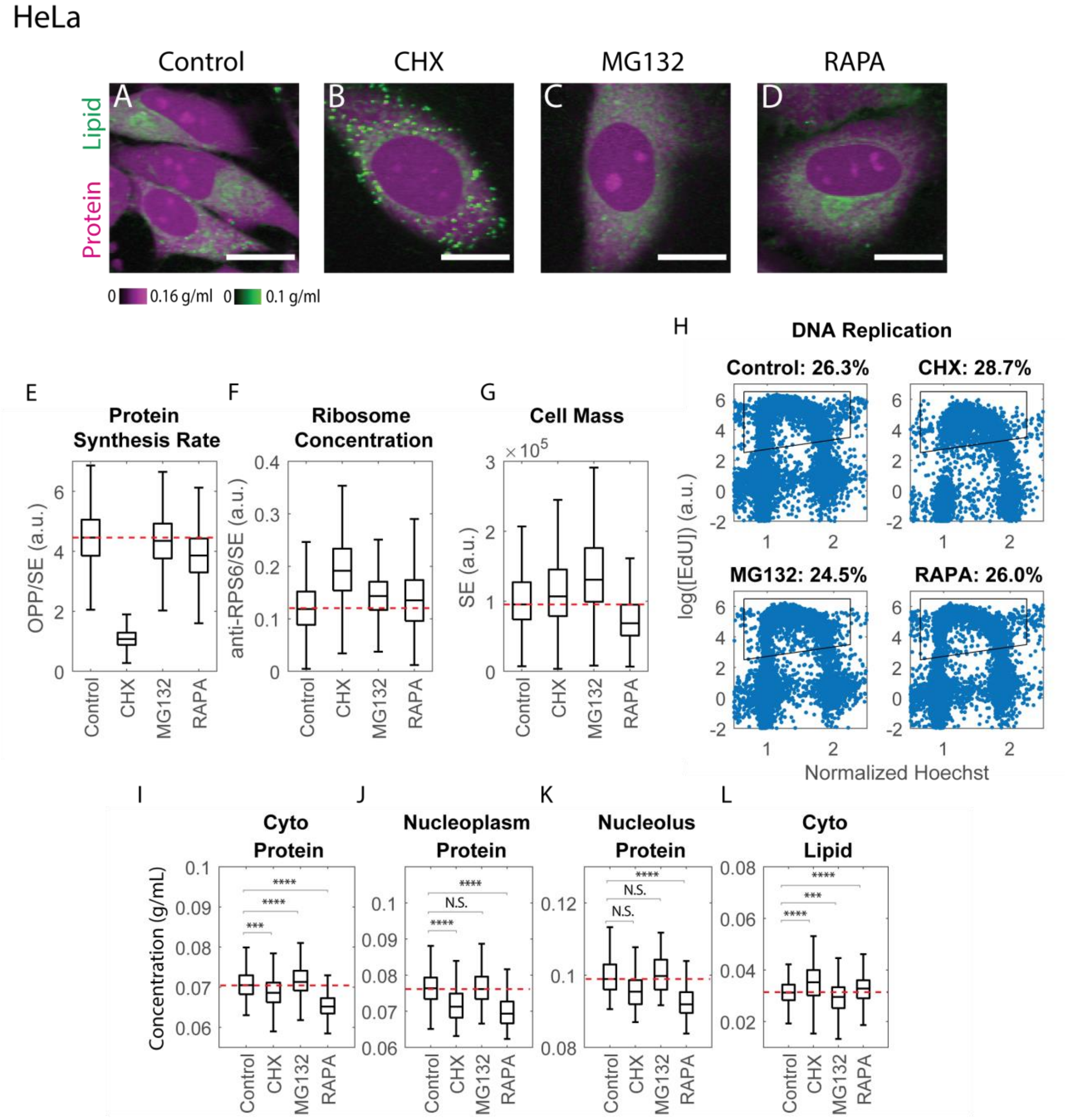

Supplementary Figure S10. Effects of perturbing protein synthesis and degradation on HeLa cell mass density. (A-D) Representative NoRI images of HeLa cells cultured in 10 µM Cycloheximide (CHX), 10 µM MG132, or 100 nM Rapamycin (RAPA) for 24 hours. Scale bar, 20 µm. (E) Protein synthesis rates quantified by the OPP pulse label to SE protein stain ratio (OPP/SE). (F) Ribosome concentration quantified by the anti-RPS6 immunostian to SE protein stain ratio (anti-RPS6/SE). (G) Cell dry mass measured by SE fluorescence. Red dashed lines in (E-G) indicate the median of control. (H) DNA replication quantified by the percentage of EdU labeled cells. The X-axis is the nuclear Hoechst intensity normalized by the highest peak of Hoechst distribution. The Y-axis is the logarithm of mean intensity of EdU in the nucleus. Each blue dot is a cell. The black outlines are the gates for EdU labeled cells. (I-L) NoRI measurement of protein density in cytoplasm, nucleoplasm, nucleolus, and lipid density in cytoplasm. N=766-840 (Control), 156-207 (CHX), 225-291 (MG132), 370-415 (RAPA).

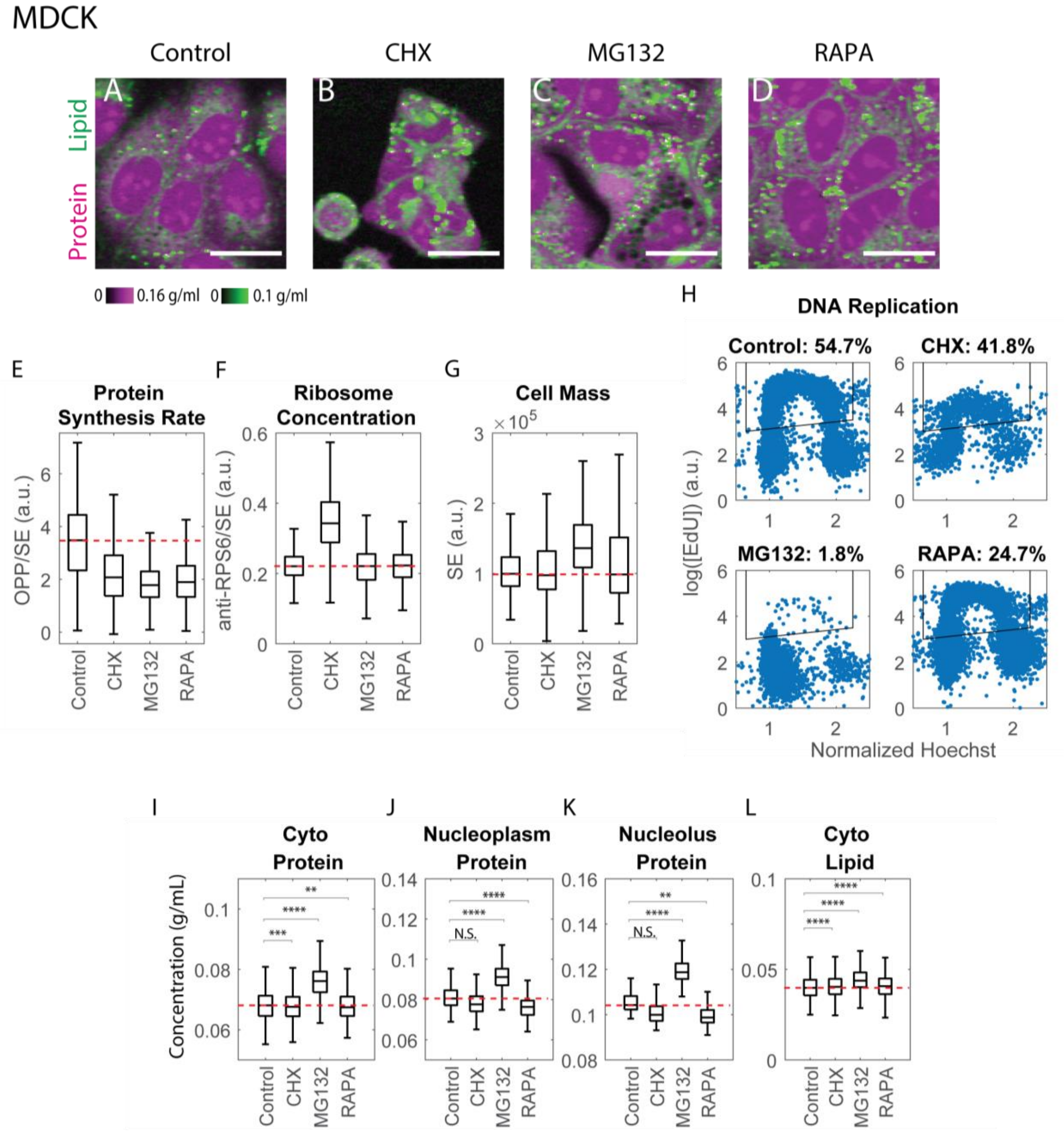

Supplementary Figure S11. Effects of perturbing protein synthesis and degradation on MDCK cell mass density. (A-D) Representative NoRI images of MDCK cells cultured in 100 nM Cycloheximide (CHX), 10 µM MG132, or 100 nM Rapamycin (RAPA) for 24 hours. Scale bar, 20 µm. (E) Protein synthesis rate quantified by the OPP pulse label to SE protein stain ratio (OPP/SE). (F) Ribosome concentration quantified by the anti-RPS6 immunostian to SE protein stain ratio (anti-RPS6/SE). (G) Cell dry mass measured by SE fluorescence. Red dashed lines in (E-G) indicate the median of control. (H) DNA replication quantified by the percentage of EdU labeled cells. The X-axis is the nuclear Hoechst intensity normalized by the highest peak of Hoechst distribution. The Y-axis is the logarithm of mean intensity of EdU in the nucleus. Each blue dot is a cell. The black outlines are the gates for EdU labeled cells. (I-L) NoRI measurement of protein density in cytoplasm, nucleoplasm, nucleolus, and lipid density in cytoplasm. N=977-1036 (Control), 232-621 (CHX), 231-350 (MG132), 397-462 (RAPA).

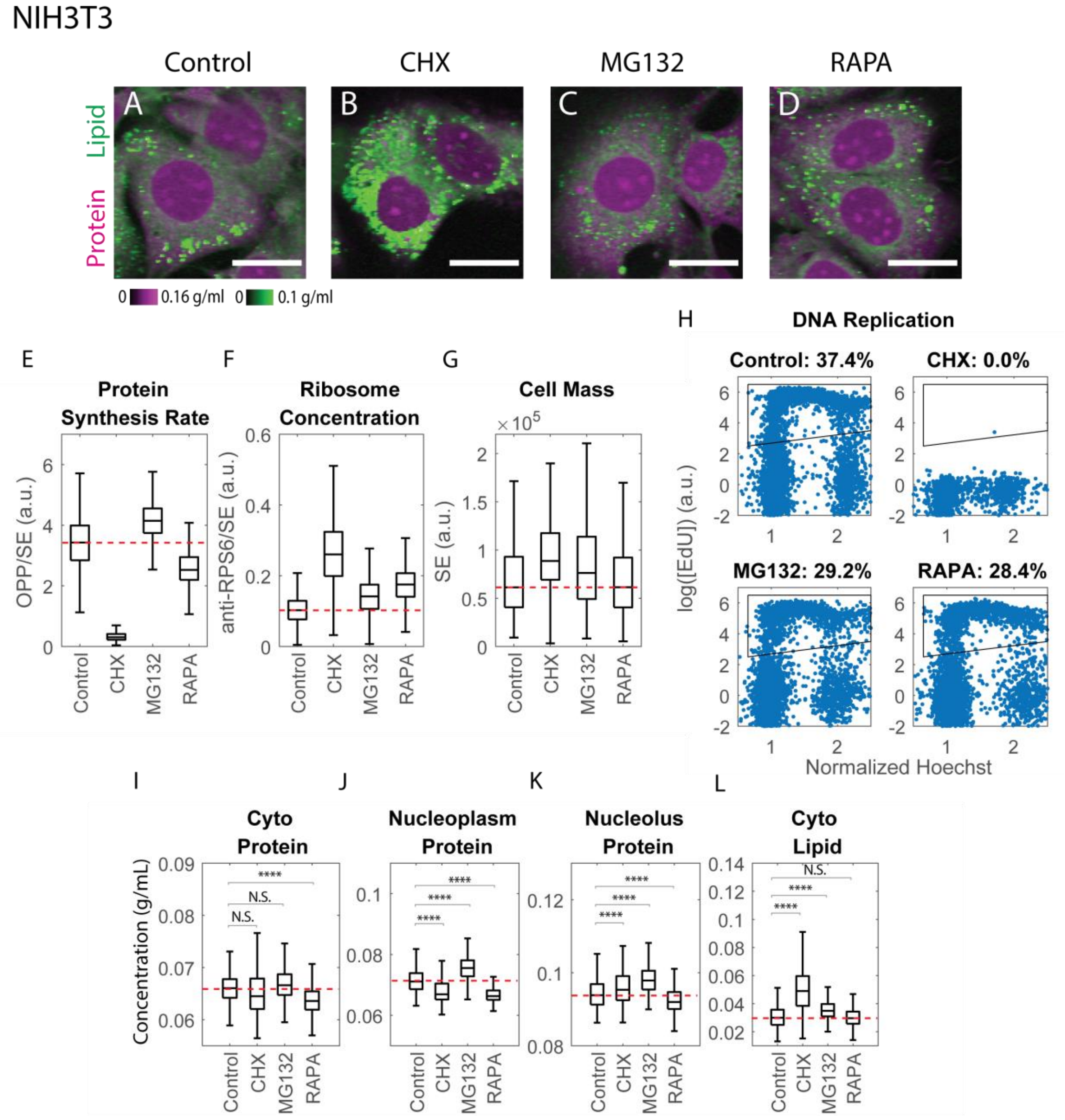

Supplementary Figure S12. Effects of perturbing protein synthesis and degradation on NIH3T3 cell mass density. (A-D) Representative NoRI images of NIH3T3 cells cultured in 10 µM Cycloheximide (CHX), 10 µM MG132, or 100 nM Rapamycin (RAPA) for 24 hours. Scale bar, 20 µm. (E) Protein synthesis rate quantified by the OPP pulse label to SE protein stain ratio (OPP/SE). (F) Ribosome concentration quantified by the anti-RPS6 immunostian to SE protein stain ratio (anti-RPS6/SE). (G) Cell dry mass measured by SE fluorescence. Red dashed lines in (E-G) indicate the median of control. (H) DNA replication quantified by the percentage of EdU labeled cells. The X-axis is the nuclear Hoechst intensity normalized by the highest peak of Hoechst distribution. The Y-axis is the logarithm of mean intensity of EdU in the nucleus. Each blue dot is a cell. The black outlines are the gates for EdU labeled cells. (I-L) NoRI measurement of protein density in cytoplasm, nucleoplasm, nucleolus, and lipid density in cytoplasm. N=585-722 (Control), 156-275 (CHX), 385-420 (MG132), 250-273, 64-75 (RAPA).

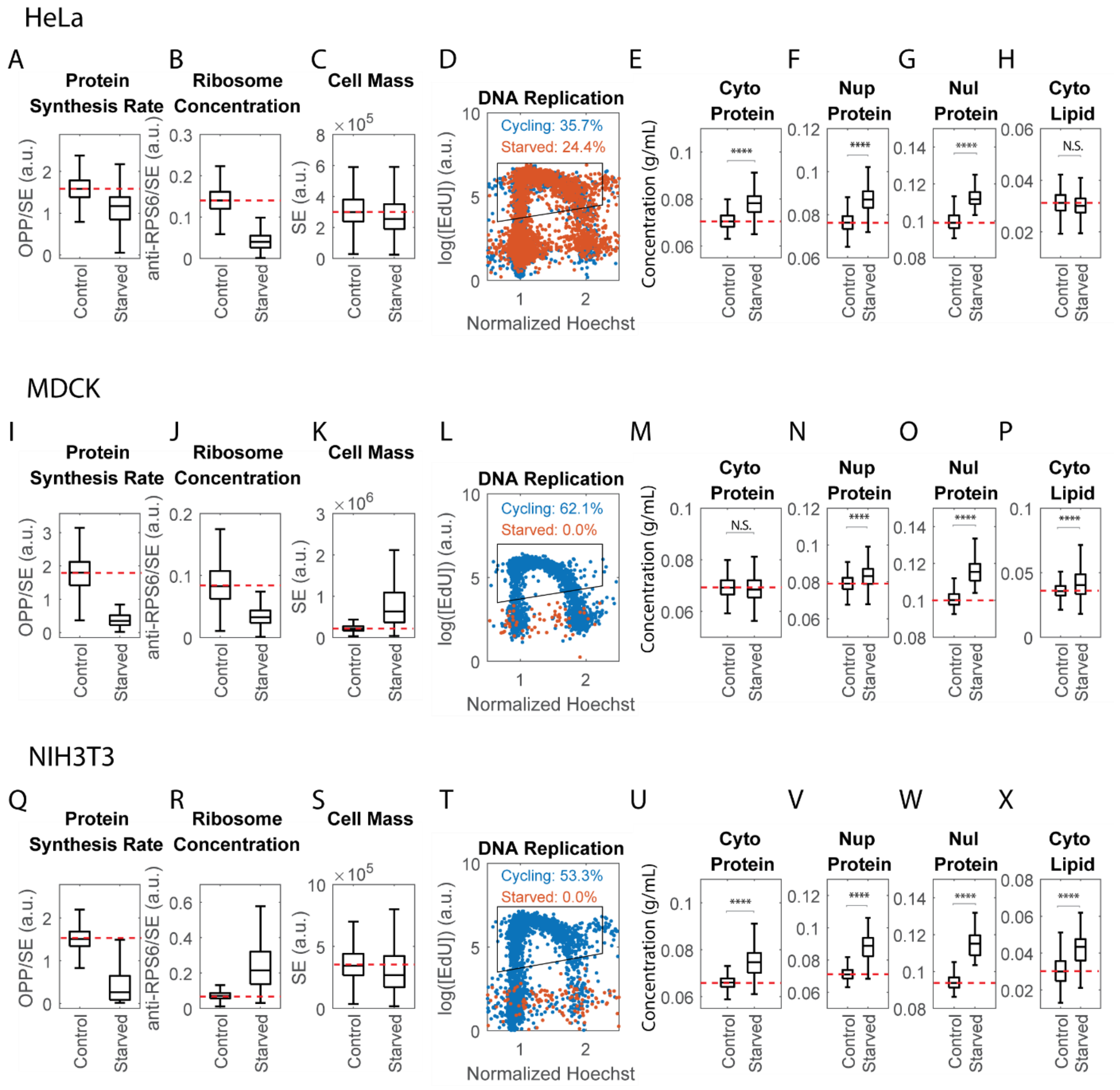

Supplementary Figure S13. Effects of starvation on cell physiology and mass density. (A, I, Q) Protein synthesis rate quantified by the ratio of pulse-labeled OPP to SE protein stain (OPP/SE). (B, J, R) Ribosome concentration quantified by the ratio of anti-RPS6 immunostain to SE protein stain (anti-RPS6/SE). (C, K, S) Cell dry mass measured by SE fluorescence. (D, L, T) DNA replication quantified by the percentage of EdU labeled cells. The X axis is the nuclear Hoechst intensity normalized by the highest peak of Hoechst distribution. The Y axis is the logarithm of mean intensity of EdU in the nucleus. Each blue or orange dot is a cell. The black outlines are the gates for EdU labeled cells. Legends indicate the percentage of cells in the gate. (E-H, M-P, U-X) NoRI measurement of protein density in cytoplasm, nucleoplasm, nucleolus, and lipid density in cytoplasm of HeLa (N=766-840 (Control), N=744-841 (Starved)), MDCK (N=977-1036 (Control), N=222-285 (Starved)), and NIH3T3 (N=585-722 (Control), N=64-75 (Starved)) cells. Red dashed lines indicate the median of control.
